# E-Health Intervention for Fear of Cancer Recurrence: A Randomized Clinical Trial

**DOI:** 10.1001/jamanetworkopen.2025.42112

**Published:** 2025-11-11

**Authors:** Johanne Dam Lyhne, Allan “Ben” Smith, Signe Timm, Britt Klein, Belinda Thewes, Afaf Girgis, Adeola Bamgboje-Ayodele, Lisa Beatty, Joanna Fardell, Per Fink, Phyllis Butow, Lisbeth Frostholm, Lars Henrik Jensen

**Affiliations:** 1 Department of Clinical Oncology, University Hospital of Southern Denmark, Vejle, Denmark; 2 Institute for Regional Health Research, University of Southern Denmark, Odense, Denmark; 3 The Daffodil Centre, The University of Sydney, A Joint Venture With Cancer Council NSW, Sydney, Australia; 4 South West Sydney Clinical Campuses, UNSW Medicine & Health, University of New South Wales, Liverpool, Australia; 5 Health Innovation & Transformation Centre, Federation University Australia, Ballarat, Australia; 6 Biopsychosocial and eHealth Research & Innovation Hub, Federation University Australia, Ballarat, Australia; 7 Rural Clinical School, School of Medicine and Psychology, Australian National University, Canberra, Australia; 8 Discipline of Design, Sydney School of Architecture, Design and Planning, University of Sydney, Sydney, Australia; 9 Flinders University Institute of Mental Health & Wellbeing, College of Education, Psychology & Social Work, Flinders University, Adelaide, Australia; 10 Psycho-Oncology Cooperative Research Group, Sydney, Australia; 11 Behavioural Sciences Unit, School of Clinical Medicine, University of New South Wales, Sydney, Australia; 12 Clinic for Functional Disorders, Aarhus University Hospital, Aarhus, Denmark; 13 Department of Clinical Medicine, Aarhus University, Aarhus, Denmark; 14 School of Psychology, University of Sydney, Sydney, Australia

## Abstract

**Importance:**

Despite the availability of effective interventions, fear of cancer recurrence (FCR) remains a prevalent concern among cancer survivors, underscoring the need for approaches that balance accessibility (eg, an online program with asynchronous therapist guidance) with engagement and effectiveness.

**Objective:**

To evaluate the efficacy of a 10-week therapist-guided (TG) e-health intervention, TG-iConquerFear, to reduce FCR in colorectal cancer (CRC) survivors.

**Design, Setting, and Participants:**

This single-site, Danish population–based randomized clinical trial enrolled cancer-free adult CRC survivors aged 18 years or older from May 8, 2023, to April 8, 2024, with clinical FCR (FCR Inventory–Short Form [FCRI-SF] score ≥22). Eligible participants had completed curative-intent CRC treatment between March 1, 2014, and December 31, 2018. Follow-up was 6 months.

**Interventions:**

Participants were randomized 1:1 (stratified by age and sex) to receive TG-iConquerFear or augmented control. TG-iConquerFear comprised 6 modules with written therapist guidance delivered remotely, individually, and asynchronously as needed over 10 weeks. Augmented control was a diagnostic interview and referral to a webpage with self-help mindfulness exercises.

**Main Outcomes and Measures:**

The primary outcome was change in FCRI total score at 3 months postintervention, analyzed according to intention-to-treat.

**Results:**

Of 9946 eligible CRC survivors, 103 with clinically significant FCR were randomized. Prior to the intervention, 2 were excluded due to cancer and 6 dropped out. Of the remaining 95 participants (median age, 63 [IQR 57-72] years; 60 [63%] female; median time since diagnosis, 7 [IQR 6-9] years), 42 were analyzed in the TG-iConquerFear group and 53 in the augmented control group. Intervention participants completed a mean (SD) of 4.5 (1.9) of 6 modules. Total FCRI score decreased significantly from baseline to 3 months in the TG-iConquerFear group (mean change, −21.7 [95% CI, −30.1 to −13.3] points) but not in the control group (mean change, −2.6 [95% CI, −7.8 to 2.6] points). This represents a between-group difference of 19.1 (95% CI, 10.0-28.3) points (*P* < .001) and a standardized effect size (Cohen *d*) of 0.62 (95% CI, 0.13-1.10). A significantly higher proportion of TG-iConquerFear participants had FCRI-SF scores below the cutoff for clinical FCR at 3 months postintervention compared with self-help participants (22 of 27 [81%] vs 18 of 42 [43%]; *P* = .002). The number needed to treat was 3.

**Conclusions and Relevance:**

In this randomized clinical trial, TG-iConquerFear intervention participants reported a significant and clinically meaningful reduction in FCR compared with the control group, suggesting an e-health intervention may expand the range of FRC treatment options beyond face-to-face interventions.

**Trial Registration:**

ClinicalTrials.gov Identifier: NCT04287218

## Key Points

**Question:**

Can a 10-week therapist-guided e-health intervention reduce the severity of fear of cancer recurrence, measured by the Fear of Cancer Recurrence Inventory (FCRI), in long-term colorectal cancer survivors 5 to 10 years postdiagnosis?

**Findings:**

In this randomized clinical trial including a nationwide Danish cohort of 95 participants, the mean FCRI score decreased significantly in the intervention but not in the control group, representing a significant between-group difference.

**Meaning:**

The findings suggest the therapist-guided e-health intervention can significantly reduce fear of cancer recurrence among long-term colorectal cancer survivors.

## Teaser

This randomized clinical trial examines the efficacy of a therapist-guided e-health intervention to reduce fear of cancer recurrence in a Danish cohort of colorectal cancer survivors.

## Introduction

Fear of cancer recurrence (FCR), defined as “fear, worry or concern about cancer returning or progressing,”^1^ is a common late effect among cancer survivors, with clinical levels reported in approximately 20% of cancer survivors generally.^2^ In colorectal cancer (CRC) survivors, clinical levels of FCR have been reported in 10% to 16%.^2,3,4^ For some CRC survivors, FCR seems enduring and has been reported up to 10 years postdiagnosis in a small subset of survivors.^5^ Clinical levels of FCR can be highly debilitating and are associated with several adverse health outcomes, including anxiety, depression, health anxiety, tiredness, CRC-specific symptom burden, and impaired overall health-related quality of life (HRQOL).^5,6^ High levels of FCR have also been associated with increased health care utilization.^7,8,9^ FCR is thus a considerable burden at both the individual and societal level, and intervention models facilitating greater access to FCR treatment are a top international research priority.^10^

During the past 3 decades, various interventions targeting FCR have been developed and evaluated.^11,12,13^ Traditional face-to-face therapy has been complemented with e-health formats, such as the adaptation of the efficacious therapist-delivered ConquerFear intervention^14,15^ into an e-health self-guided format (iConquerFear). E-health has the possibility to overcome both recipient-related barriers of accessing support, such as traveling distance, time constraints, and disease burden, and clinician-related barriers, such as lack of qualified clinicians and time. Although more robust evidence is needed,^16^ guided digital interventions with guidance delivered via e-consultations (eg, video or telephone calls) have shown outcomes comparable to face-to-face interventions in reducing FCR.^11,17^ However, whether such guidance is efficacious when delivered in a written and asynchronous format remains unknown.

The efficacy of self-guided e-health interventions can be limited by low uptake and engagement,^18^ as seen with iConquerFear.^14^ According to ConquerFear therapists, online delivery was seen as a way to increase intervention reach but should ideally not be unguided.^19^ To address this, iConquerFear was further adapted into a therapist-guided (TG) format (TG-iConquerFear)^20^ to capitalize on the therapeutic relationship with a health professional achieved in face-to-face settings but also the flexibility and scalability of e-health.

This trial aimed to evaluate the efficacy of TG-iConquerFear, a written, asynchronous, therapist-guided e-health intervention, in a population-based cohort of CRC survivors reporting clinical levels of FCR 5 to 10 years postdiagnosis. The intervention group was compared with an augmented control group receiving diagnostic interview and referral to a webpage with self-help mindfulness exercises. We hypothesized that TG-iConquerFear participants would experience larger reductions in FCR compared with augmented control participants at 3 months postintervention.

## Methods

### Study Design and Participants

Conduct and reporting of this randomized clinical trial adhered to the Consolidated Standards of Reporting Trials (CONSORT) reporting guideline^21^ and published protocol.^22^ In short, adult (aged ≥18 years) Danish cancer-free CRC survivors who had completed curative-intent CRC treatment between March 1, 2014, and December 31, 2018, were identified through the Danish Colorectal Cancer Group (DCCG) database and invited to complete an 86-item electronic survey assessing late effects, including FCR. CRC survivors with clinically significant FCR (defined as a Fear of Cancer Recurrence Inventory–Short Form [FCRI-SF] score ≥22 on a scale of 0 to 36, with higher scores indicating worse FCR^23,24,25^) were automatically asked if they were interested in engaging in FCR treatment. All eligible participants underwent a brief semistructured diagnostic interview^26,27^ by telephone to confirm suitability. Participants with urgent psychological needs (eg, severe depression) were excluded and advised to seek appropriate professional care. Consenting participants were randomized 1:1 to TG-iConquerFear or augmented control by a random allocation sequence generated by REDCap and stratified by age and sex. This study was performed in line with the principles of the Declaration of Helsinki.^28^ Ethical approval was obtained from the Regional Committees on Health Research Ethics for Southern Denmark. All participants provided oral and written informed consent prior to participation. The trial protocol can be found in Supplement 1.

### Intervention and Augmented Control

TG-iConquerFear is an e-health intervention delivered as a manual comprising 6 modules with written, asynchronous therapist support.^20,29^ The complete ConquerFear curriculum and manual are available online.^30^ The modules focus on values-based goal setting, fostering acceptance of uncertainty, strategies to control worry, challenging unhelpful beliefs, and developing appropriate monitoring behaviors.

Guidance was delivered by licensed psychologists with over 4 years of experience in online therapy. Guidance was provided asynchronously based on the ConquerFear manual over a period of 10 weeks through an embedded messaging system. Therapists had full access to all activities completed by the participant within the program, enabling tailored support. In case of inactivity for a period of more than 3 weeks, the therapists were instructed to contact the participant via telephone or text message. In case of adverse effects, the primary investigator (J.D.L.) was informed.

Participants allocated to the control condition were referred to a public website with informational videos on cancer survivorship care and self-guided meditation exercises.^31^ Website use could not be monitored. All participants had lifelong access to publicly funded general practitioners and partially subsidized specialist care, including psychological services.

### Measures

Demographic and clinical data were obtained through the DCCG database. Participants completed primary, secondary, and process outcome measures at baseline (T0), at 2 weeks postintervention for intervention participants or 12 weeks postrandomization for augmented control participants (T1), and 3 months (T2) and 6 months (T3) after each group’s T1.

#### Primary Outcome

FCR during the intervention period was assessed using the Danish version of the 42-item FCRI.^4^ The FCRI includes subscales evaluating FCR triggers, psychological distress, severity, functional impairments, insights, reassurance, and coping strategies^23^ on a 5-point Likert-like scale ranging from 0 (“not at all” or “never”) to 4 (“a great deal” or “all the time”). Total FCRI scores range from 0 to 168; higher scores indicate worse FCR. The FCRI has demonstrated internal consistency, satisfactory construct validity, temporal stability, and responsiveness to change.^4,23^ The primary trial outcome was predefined as change in FCRI total score at 3 months postintervention (T2), as the total FCRI score assesses dimensions of FCR more closely aligned with agreed-upon characteristics of clinical FCR.^32^

#### Secondary Outcomes

Anxiety, depression, and emotional distress were evaluated with the 13-item subscale of the Symptom Checklist-90-Revised,^33,34^ HRQOL with the visual analogue scale (VAS) from the EuroQol 5-Dimension 5-Level questionnaire,^35^ and physical symptom burden with the Bodily Distress Syndrome Checklist.^36^ Additionally, change in total FCRI score at T1 and T3 along with FCRI subscale scores were predefined as secondary outcomes.

#### Process Outcomes

Negative beliefs about worry were assessed using a subscale of the MetaCognitions Questionnaire 30,^37^ while perceived risk of recurrence was measured by a VAS from 1 to 100, with higher scores indicating higher perceived risk of recurrence.^38^ Process measures were assessed in the intervention group at baseline, in modules 3 and 5, and at T1 through T3.

#### Adherence and Engagement Metrics

Participant adherence was assessed by the number of modules completed (range, 0-6). The amount of therapist guidance was measured by the number and length of messages. Participant engagement was evaluated by the therapist on a scale from 0 (not engaged) to 10 (highly engaged with all elements of the intervention and feedback), based on the therapist’s subjective impression.

### Sample Size

A conservative a priori statistical sample size calculation indicated that a minimum of 246 participants would be required to detect a standardized mean difference in total FCRI scores (Cohen *d* of 0.5, with a group difference of 3.5 and an SD of 7.0) with 90% power and 2-sided α = .05, assuming a 30% dropout rate.^22^ The lower-than-expected prevalence of clinical FCR^5^ limited the eligible sample. Meanwhile, comparable trials have demonstrated robust effects in samples of fewer than 100 participants,^39,40,41^ supporting the adequacy of the achieved sample size.

### Statistical Analysis

Baseline demographic and cancer-related group characteristics were compared using balance diagnostics and reported as standardized differences accompanied by descriptive statistics. An absolute standardized difference greater than 0.2 was interpreted as imbalance between groups.^42^

Unadjusted changes in primary and secondary outcomes from baseline to each follow-up were compared pairwise using paired *t* tests. Change scores were calculated based on complete pre-post matched data within each group. Between-group comparisons were then based on these individual change scores, using an independent-samples *t* test. Cohen *d* was calculated to assess the effect size of the between-group differences in the primary outcome from baseline to follow-up. Conventionally, values of 0.2, 0.5, and 0.8 are interpreted as small, medium, and large effect sizes, respectively.^43^ Adherence and engagement metrics were analyzed using descriptive statistics, including medians (IQRs) and frequency distributions, due to the skewed distribution of data.

Proportions of participants above or below the FCRI-SF clinical cutoff score (≥22)^24^ were analyzed using χ^2^ tests as intention-to-treat. Per-protocol analyses were not performed, since participants classified as passive users (ie, completing less than 2 modules) were lost to follow-up without contributing data to the results of primary or secondary outcomes. In addition to continuous analyses of the primary outcome, the number needed to treat (NNT) was calculated using a binary outcome based on the validated FCRI-SF cutoff score of 22,^24^ which facilitates clinical interpretation. Exploratory post hoc analyses compared characteristics of completers, who completed all 6 modules, with noncompleters completing 2 to 5 modules.

Missing items on measures were replaced with a 0, assuming that the symptom in question was not present. Replacement of missing items with 0 was only applied at baseline; follow-up questionnaires were programmed as mandatory, and therefore, no item-level missingness occurred at follow-up. If more than half of items were missing, the sum score was not calculated. No imputation was performed.^44^ Two-sided *P* values less than .05 were considered statistically significant. Statistical analyses were performed using Stata, version 18 (StataCorp LLC).

## Results

**Figure 1.**
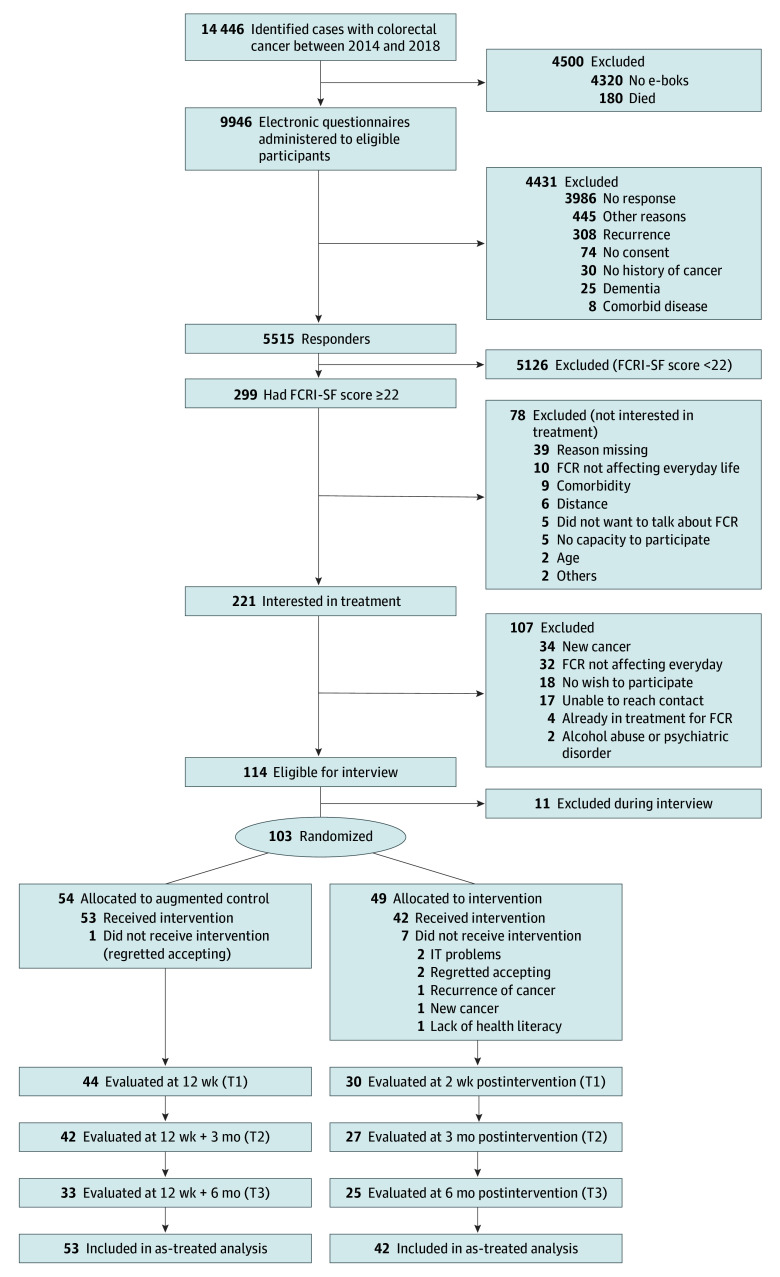
CONSORT Diagram e-Boks is a secure national digital mailbox system used in Denmark for communication between citizens, companies, and public authorities. FCR indicates fear of cancer recurrence; FCRI-SF, Fear of Cancer Recurrence Inventory–Short Form; IT, information technology.

Of 9946 eligible CRC survivors invited between May 8, 2023, and April 8, 2024, 5515 (55%) completed the FCR screening, and 299 of those (5%) scored at or above the FCRI-SF cutoff of 22, indicating clinical FCR. As shown in Figure 1, 221 of those (74%) were interested in treatment for FCR. Main reasons reported for not being interested were FCR not affecting everyday life (n = 10) or comorbidity (n = 9). During the informed consent process, 107 of the interested CRC survivors (48%) were excluded, mainly due to new cancer diagnoses (n = 34) or FCR not affecting everyday life (n = 32). Diagnostic interview with the remaining 114 CRC survivors led to the exclusion of 11 (10%), mainly due to urgent mental health concerns. Of the remaining 103 survivors (34% of those meeting the threshold for clinical FCR), 49 were randomized to TG-iConquerFear and 54 to augmented control. Two participants from the intervention group and 1 from the control group were excluded after randomization due to regretting acceptance, and 5 additional participants from the intervention group dropped out due to new or recurring cancer, information technology problems, or lack of health literacy. In total, 95 CRC survivors (42 randomized to TG-iConquerFear and 53 to augmented control) were included in the final analysis (median age, 63 years [IQR, 57-72 years]; 60 [63%] female, 35 [37%] male; median of 7 years [IQR, 6-9 years] since diagnosis).

As seen in Figure 1, 26 participants (27%) were lost to follow-up at 3 months postintervention (primary outcome), 15 (36%) from the TG-iConquerFear group and 11 (21%) from the augmented control group. Group allocation, baseline FCRI-SF score, sex, and age were not associated with loss to follow-up. Minor differences were seen in participant characteristics between the 2 groups (Table 1). Intervention group participants reported a more recent cancer-related appointment at the hospital compared with the control group and were more highly educated. Mean (SD) baseline FCRI score was 84.6 (18.9) in the intervention and 84.6 (16.5) in the control group.

**Table 1.** Patient Characteristics

Characteristic	Participants^a^			Standardized difference^b^
	Total (N = 95)	Intervention (n = 42)	Augmented control (n = 53)	
Age, median (IQR), y	63 (57-72)	65 (58-72)	63 (56-70)	−0.30
Sex				
Female	60 (63)	27 (64)	33 (62)	0.04
Male	35 (37)	15 (36)	20 (38)	
Cancer type				
Colon	62 (65)	27 (64)	35 (66)	0.04
Rectum	33 (35)	15 (36)	18 (34)	
Cancer stage at time of diagnosis				
Nonmetastatic	88 (96)	41 (98)	47 (87)	0.24
Metastatic	6 (6)	1 (2)	5 (9)	
Unknown	1 (1)	0	1 (2)	
Time since diagnosis, median (IQR), y	7 (6-9)	7 (6-8)	7 (6-9)	−0.06
Time since most recent appointment				
>1 y	55 (58)	24 (57)	31 (58)	0.35
3-12 mo	31 (33)	11 (26)	20 (38)	
1 wk to 3 mo	9 (9)	7 (17)	2 (4)	
<1 wk	0	0	0	
Time to next appointment				
<1 wk	2 (2)	2 (5)	0	0.55
1 wk to 3 mo	10 (11)	4 (19)	6 (11)	
3-12 mo	9 (9)	3 (7)	6 (11)	
>1 y	39 (41)	17 (40)	22 (42)	
Cancer control ended	35 (37)	16 (38)	19 (36)	
Chemotherapy received	53 (56)	26 (62)	27 (51)	0.22
Radiotherapy received	7 (7)	2 (5)	5 (9)	0.18
Living status				
With others	65 (68)	31 (74)	34 (64)	0.20
Alone	30 (32)	11 (26)	19 (36)	
Employment status				
Employed	45 (47)	19 (45)	26 (49)	0.23
Has been employed	48 (51)	22 (52)	26 (49)	
Never employed	1 (1)	1 (2)	0	
Not reported	1 (1)	0	1 (2)	
Level of education				
Short (<10 y)	14 (15)	7 (17)	7 (13)	0.54
Medium (10-12 y)	42 (44)	13 (31)	29 (55)	
High (>12 y)	34 (36)	20 (48)	14 (26)	
Military career	3 (3)	1 (2)	2 (4)	
Missing	2 (2)	1 (2)	1 (2)	
Citizenship				
Danish	90 (95)	40 (95)	50 (94)	0.04
Not Danish	4 (4)	2 (5)	2 (4)	
Not reported	1 (1)	0	1 (2)	
Children				
Yes	89 (94)	39 (93)	50 (94)	0.06
No	6 (6)	3 (7)	3 (6)	
Participated in CRC screening				
Yes	22 (23)	9 (21)	13 (25)	0.07
No	73 (77)	33 (79)	40 (75)	

Abbreviation: CRC, colorectal cancer.

^a^ Data are reported as number (percentage) of participants unless otherwise indicated.

^b^ Standardized differences greater than 0.20 were considered indicative of imbalance between groups.

### Primary Outcome

Total FCRI score significantly decreased from T0 to T2 in TG-iConquerFear participants (mean change, −21.7 points; 95% CI, −30.1 to −13.3 points) but not in the augmented control group (mean change, −2.6 points; 95% CI, −7.8 to 2.6 points) (Table 2). Difference between groups at T2 was 19.1 points (95% CI, 10.0-28.3 points), corresponding to a between-group standardized effect size (Cohen *d*) of 0.62 (95% CI, 0.13-1.10).

**Table 2.** FCRI Total Score at Baseline, Posttreatment, and 3- and 6-Month Follow-Up

Time point^a^	Intervention			Augmented control			Mean difference between groups (95% CI)^c^	*P* value
	Participants, No. (%) (n = 42)	FCRI total score, mean (SD)^b^	*P* value	Participants, No. (%) (n = 53)	FCRI total score, mean (SD)^b^	*P* value		
T0	42 (100)	84.6 (18.9)	NA	53 (100)	84.6 (16.5)	NA	−0.1 (−7.3 to 7.1)	NA
T1	30 (71)	69.3 (22.4)	NA	44 (83)	79.0 (25.7)	NA	9.7 (−1.8 to 21.2)	NA
Within-group change (T0 − T1)	30 (71)	−20.1 (−27.9 to −12.3)	<.001	44 (83)	−5.5 (−11.0 to 0.4)	.07	14.6 (5.1 to 24.1)	.003
T2	27 (64)	67.1 (24.5)	NA	42 (79)	81.1 (21.1)	NA	14.0 (2.9 to 25.1)	NA
Within-group change (T0 − T2)	27 (64)	−21.7 (−30.1 to −13.3)	<.001	42 (79)	−2.6 (−7.8 to 2.6)	.33	19.1 (10.0 to 28.3)	<.001
T3	25 (59)	64.0 (24.5)	NA	33 (62)	72.5 (20.7)	NA	8.6 (−3.3 to 20.5)	NA
Within-group change (T0 − T3)	25 (59)	−24.6 (−32.7 to −16.6)	<.001	33 (62)	−6.8 (−13.0 to −0.6)	.03	17.9 (8.1 to 27.6)	.001

Abbreviations: FCRI, Fear of Cancer Recurrence Inventory; NA, not applicable.

^a^ T0 indicates baseline; T1, 2 weeks postintervention for intervention participants or 12 weeks postrandomization for augmented control participants; T2, 3 months after each group’s T1; and T2, 6 months after each group’s T1.

^b^ Score range 0 to 168, with higher scores indicating worse fear of cancer recurrence.

^c^ The change score is based on complete matched data within the group, not the difference between the group’s total mean score at each time point. Hence, differences between groups are based on completed matched data within groups.

### Secondary Outcomes

**Figure 2.**
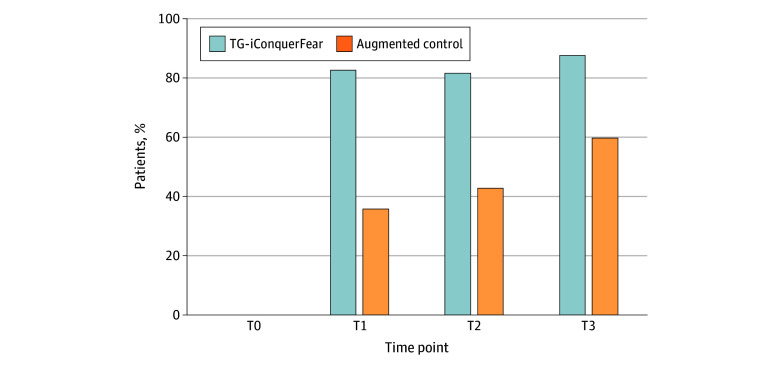
Percentages of Patients Below the Cutoff for Clinical Fear of Cancer Recurrence (FCR) Clinical FCR was defined as a score of 22 or higher on the FCR Inventory–Short Form (score range, 0-36, with higher scores indicating worse FCR). T0 indicates baseline; T1, 2 weeks postintervention for intervention participants or 12 weeks postrandomization for augmented control participants; T2, 3 months after each group’s T1; and T2, 6 months after each group’s T1. As having clinical FCR was an inclusion criterion, no participants are shown in the figure at T0. TG indicates therapist-guided.

Between-group difference in FCRI total score at T3 was 17.9 points (95% CI, 8.1-27.6 points) (Table 2), with a Cohen *d* of 0.38 (95% CI, −0.14 to 0.90). Across all follow-up time points, significantly more TG-iConquerFear participants had FCRI-SF scores below the cutoff for clinical FCR (ie, <22) compared with augmented control participants (eg, 22 of 27 [81%] vs 18 of 42 [43%] at 3 months postintervention; *P* = .002) (Figure 2). NNT was 3. Statistically significant between-group differences were seen in levels of FCR severity, anxiety, depression, emotional distress, quality of life, and burden of physical symptoms in both groups over time (Table 3), except for depression at T3 (*P* = .08). TG-iConquerFear participants showed significantly greater improvement than augmented control participants in 4 out of 6 subscales of the FCRI (triggers, psychological distress, functional impairments, and insight) but not in reassurance and coping strategies (eTables 1-6 in Supplement 2).

**Table 3.** Change in Outcomes From Baseline to 3-Month and 6-Month Follow-Up Assessments

Outcome (score range)^a^	Time point^b^						Mean difference between groups at T2 (95% CI)^c^	*P* value	Mean difference between groups at T3 (95% CI)^c^	*P* value
	T0		T2		T3					
	Participants, No./total No. (%)	Mean (SD)	Participants, No./total No. (%)	Mean (SD)	Participants, No./total No. (%)	Mean (SD)				
**FCRI-SF (0-36)**										
Intervention	42/42 (100)	25.4 (2.9)	27/42 (64)	16.6 (5.3)	25/42 (60)	16.6 (6.3)	6.5 (4.2-8.8)	<.001	5.8 (3.4-8.2)	<.001
Control	53/53 (100)	24.8 (1.9)	42/53 (79)	22.0 (4.8)	33/53 (62)	20.6 (4.0)				
**Anxiety (0-16)**										
Intervention	42/42 (100)	4.9 (3.5)	27/42 (64)	3.0 (2.8)	25/42 (60)	2.6 (2.9)	2.3 (0.7-3.8)	.004	2.4 (0.8-4.0)	.004
Control	52/53 (98)	5.0 (3.5)	41/53 (77)	4.6 (3.3)	32/53 (60)	4.1 (3.2)				
**Depression (0-24)**										
Intervention	40/42 (95)	5.1 (4.6)	27/42 (64)	3.2 (2.9)	25/42 (60)	3.4 (3.7)	2.3 (0.2-4.5)	.04	2.1 (−0.2-4.5)	.08
Control	51/53 (96)	5.6 (5.1)	41/53 (77)	5.0 (4.4)	32/53 (60)	4.7 (4.6)				
**Emotional distress (0-32)**										
Intervention	42/42 (100)	9.5 (6.1)	27/42 (64)	6.1 (5.1)	25/42 (60)	5.7 (5.1)	4.3 (1.0-7.6)	.01	3.9 (0.6-7.2)	.02
Control	51/53 (96)	10.2 (6.8)	41/53 (77)	9.3 (6.9)	32/53 (60)	8.4 (5.3)				
**Quality of life (0-100)**										
Intervention	42/42 (100)	61.0 (20.4)	26/42 (62)	69.5 (17.2)	24/42 (67)	73.7 (17.5)	8.5 (0.4 to 16.6)	.04	8.2 (0.1-16.3)	.047
Control	53/53 (100)	60.9 (23.0)	42/53 (79)	62.0 (21.8)	33/53 (62)	64.3 (20.7)				
**Physical symptoms (0-100)**										
Intervention	37/42 (88)	31.8 (17.8)	27/42 (64)	25.2 (18.5)	25/42 (60)	24.2 (17.1)	10.2 (2.5-17.9)	.01	12.1 (4.0-20.1)	.004
Control	40/53 (75)	26.7 (17.4)	41/53 (77)	28.0 (15.3)	32/53 (60)	27.2 (16.4)				

Abbreviation: FCRI-SF, Fear of Cancer Recurrence Inventory–Short Form.

^a^ Higher scores indicate worse outcomes.

^b^ T0 indicates baseline. T1 was 2 weeks postintervention for intervention participants or 12 weeks postrandomization for augmented control participants; T2 and T3 are 3 months and 6 months, respectively, after each group’s T1.

^c^ The change score is based on complete matched data within the group, not the difference between the group’s total mean at each time point. Hence, differences between groups are based on completed matched data within groups.

### Process Measures

A decrease in the process measure of negative beliefs about worry (from a mean [SD] of 12.0 [3.7] to 10.1 [2.7] on a scale from 0-24, with higher scores indicating more negative beliefs about worry; *P* = .01) and perceived risk of recurrence (from 55.7 [18.2] to 42.3 [23.0] on a scale from 0-100, with higher scores indicating higher perceived risk of recurrence; *P* = .03) was also observed at week 8 (module 5). Levels of negative beliefs about worry and of perceived recurrence risk remained stable throughout the follow-up period (eTable 7 in Supplement 2).

### Adherence and Engagement Metrics

The mean (SD) adherence was completion of 4.5 (1.9) modules. A total of 23 participants in the intervention group (55%) completed all 6 modules. Five participants completed only module 1 and were classified as passive users, corresponding to a dropout rate of 12%. All but 4 (17%) of the participants completing all 6 modules had complete follow-up data. Participants received a median of 12 messages (range, 2-29 messages). The median message length was 139 words (range, 70-220 words). Participants with a higher HRQOL score and those who actively communicated with their therapist were more likely to complete a greater number of modules. No other characteristics of completers were observed (eTable 8 in Supplement 2). The median baseline engagement of the 42 participants engaging in the intervention, as measured by the therapists, was 9 (IQR, 9-10) on a scale from 1 to 10 (with higher scores indicating higher engagement) and remained stable throughout the intervention period. On 23 occasions, therapists contacted participants by phone or text. Of these contacts, 20 (87%) were due to inactivity, with only 1 instance (4%) among the 23 participants who completed all 6 modules. Two contacts (9%) were related to technical issues and 1 (4%) involved further clarification of an exercise. Intervention time per participant estimated by the therapists was 20 minutes per week for the 10-week intervention. A post hoc power analysis based on the between-group difference in change in FCRI total score indicated 84% power in the complete-case sample (n = 95) and 88% when including all randomized participants (n = 103).

### Adverse Effects

One participant (2%) reported adverse effects of the intervention, as the module on self-examination (module 5) exacerbated the participant’s FCR. It gave an increased awareness of bodily symptoms and led to the diagnosis of severe endometriosis.

## Discussion

With the flexibility of asynchronous, written communication (ie, no scheduled sessions), TG-iConquerFear demonstrated clinically and statistically significant effects in reducing FCR in long-term cancer survivors compared with an augmented control condition. Our results align with those from the original face-to-face ConquerFear^15^ and ConquerFear-Group^39^ studies, with TG-iConquerFear demonstrating a larger effect size at 3 months postintervention. This difference may be attributed to the more-active control groups in the face-to-face and group trials, where control participants experienced greater reduction in FCRI total scores compared with the augmented control participants in our study. Additionally, the eligibility criterion of an FCRI-SF score of 22 or higher in our trial may have provided a larger margin for improvement compared with the original intervention, which included participants with an FCRI-SF score of 13 or higher. When compared with preliminary results from the self-guided version of iConquerFear,^14^ TG-iConquerFear achieved greater reductions in FCRI-SF at 3 months, which is consistent with a systematic review showing greater effects in guided digital interventions.^13^

TG-iConquerFear achieved reductions in FCR total scores comparable to those observed in other trials of FCR interventions combining online components with different forms of health professional support.^41,45,46^ The asynchronous, flexible, and predominantly patient-initiated support in TG-iConquerFear may facilitate the provision of care tailored to individual patient needs, which could contribute to improved outcomes.

Guidance has been identified as a key factor in enhancing both adherence and engagement,^13^ along with the relatability of the interventions content,^29^ which were central reasons for adapting iConquerFear into TG-iConquerFear. Qualitative feedback from the pilot RCT of iConquerFear suggested that a lack of guidance from health professionals was 1 of the reasons for the null effects.^47^ In the self-directed format of iConquerFear, 36% of participants completed all modules,^14^ while in TG-iConquerFear, the completion rate was 53%. Face-to-face versions of ConquerFear achieved completion rates of 63%^15^ and 67%,^39^ suggesting that greater therapeutic support is associated with improved adherence, although greater therapeutic alliance did not predict greater benefit from ConquerFear.^25^ The lack of statistically significant differences in FCR reductions between completers and noncompleters, as seen in other studies,^45^ may be due to limited statistical power due to small numbers in the group of noncompleters.

### Clinical Implications

Given the demonstrated efficacy of this accessible and scalable intervention, it could be argued that screening for FCR in routine clinical care^48^ may be beneficial for stratifying patients and providing timely, severity-matched interventions.^49,50^ TG-iConquerFear may serve as a viable alternative to or intermediate step in face-to-face interventions, thereby expanding the range of available treatment options. This strategy could optimize resource use while increasing access to psychological support, particularly for patients who are reluctant or unable to participate in in-person sessions.

Only 5% of the total cohort met the threshold for clinical FCR, and just 34% of those were included in the study. The discrepancy between survey-reported clinical FCR and the extent to which FCR affects daily life and generates a need for support underscores the importance of refining patient selection for intervention entry. Ultimately, improving identification of patients with significant need for FCR support remains a crucial objective for routine clinical practice.^51^

### Limitations

The study has limitations. This well-balanced randomized clinical trial used population-based screening of all Danish CRC survivors diagnosed over a 5- to 10-year period to identify eligible participants. All participants had equal access to the same tax-funded public health care system, minimizing selection bias. However, this context may also limit generalizability, as health care delivery, digital infrastructure, and patient characteristics may differ in countries with other models of cancer follow-up or where access to psychosocial care is less standardized. Cultural factors, such as attitudes toward psychological support and acceptance of digital health tools, together with the universal Danish health care model, may have facilitated uptake and engagement; translation of these findings to other settings will therefore require consideration of differences in health care organization, digital literacy, and socioeconomic context. All participants underwent a diagnostic interview establishing the relevance of the intervention by examining FCR and related mental disorders. The intervention and control groups were comparable in terms of demographic and clinical characteristics at baseline, strengthening the internal validity of our findings. The intervention was in the form of a manual and was delivered by experienced psychologists.

Although the a priori sample size was not reached, the study retained sufficient statistical power to detect both statistically significant and clinically meaningful effects. A post hoc power analysis based on the observed between-group difference in FCRI total score change (Cohen *d* = 0.62) indicated 84% power in the complete-case sample and 88% when including all randomized participants. These estimates suggest that the study maintained adequate statistical power to detect the observed effect.

This study’s e-health intervention was limited to participants with computer proficiency, and its applicability may be restricted to CRC survivors, as it was specifically tailored to this population.^20^ Recruiting from a registry could have negatively influenced motivation compared with recruiting through self-referral or routine clinical care.^52^ Moreover, recruiting via a survey in the digital citizen mailbox may have introduced selection bias between responders and nonresponders, with more technology-literate participants more likely to participate, though this was not explicitly demonstrated.^44^ The inclusion criteria restricted participants to those with clinically significant FCR, leaving the efficacy of the intervention for individuals with mild or moderate FCR unexamined. Additionally, attrition bias may have been introduced due to dropout, which was 12%, although a certain amount of dropout is anticipated in this kind of study. At the 3-month time point, 27% of participants were lost to follow-up. Although loss to follow-up was not associated with group allocation, FCR level, age, or sex, the resulting loss of participants may have affected the study’s statistical power. While the number of participants nonadherent to the intervention was small (n = 5), the lack of per-protocol analysis limits assessment of the robustness of the intention-to-treat results. In addition, the study was not powered to conduct subgroup analyses to identify which demographic or clinical characteristics predict greater FCR reduction.

## Conclusions

In this randomized clinical trial of the TG-iConquerFear intervention to reduce FCR in CRC survivors, intervention participants reported a significant reduction in FCR at 3 months postintervention, with sustained effects at 6 months postintervention. Anxiety, depression, emotional distress, quality of life, and burden of physical symptoms were also improved. Future research should focus on implementation studies and effectiveness in clinical settings and explore the applicability of TG-iConquerFear across different cancer types.

## Notes

Supplement 1.
Trial Protocol

Supplement 2. **eTable 1.** Change in FCRI Triggers Subscale Between Baseline (T0) and Postintervention (T1) and 3- and 6-Month Follow-Up (T2 and T3) **eTable 2.** Change in FCRI Psychological Distress Subscale Between Baseline (T0) and Postintervention (T1) and 3- and 6-Month Follow-Up (T2 and T3) **eTable 3.** Change in FCRI Functional Impairment Subscale Between Baseline (T0) and Postintervention (T1) and 3- and 6-Month Follow-Up (T2 and T3) **eTable 4.** Change in FCRI Insight Subscale Between Baseline (T0) and Postintervention (T1) and 3- and 6-Month Follow-Up (T2 and T3) **eTable 5.** Change in FCRI Reassurance Subscale Between Baseline (T0) and Postintervention (T1) and 3- and 6-Month Follow-Up (T2 and T3) **eTable 6.** Change in FCRI Coping Strategies Subscale Between Baseline (T0) and Postintervention (T1) and 3- and 6-Month Follow-Up (T2 and T3) **eTable 7.** Process Measures: Change in Outcome During Intervention Compared to Baseline **eTable 8. **Characteristics of Completers Compared to Noncompleters

Supplement 3.
Data Sharing Statement
