# Supplementary material for: E-Health Intervention for Fear of Cancer Recurrence: A Randomized Clinical Trial
Source: JAMA Netw Open. 2025 Nov 11;8(11):e2542112. doi: 10.1001/jamanetworkopen.2025.42112 (PMC12606383; doi:10.1001/jamanetworkopen.2025.42112)
Supplement: Supplement 1. — Trial Protocol [file jamanetwopen-e2542112-s001.pdf]

Psychosocial aspects of surviving colorectal cancer

A Research Protocol

Primary investigator
Johanne Dam Lyhne, MD
Department of Clinical Oncology
Vejle Hospital
Beriderbakken 4
7100 Vejle
Phone: 79406707 / 79406000
Johanne.Dam.Lyhne@rsyd.dk

Sponsor
Lars Henrik Jensen, MD, PhD, consultant
Department of Clinical Oncology
Vejle Hospital
Beriderbakken 4
7100 Vejle
Phone: 79406802 / 79406000
Lars.Henrik.Jensen@rsyd.dk

Collaborators
Per Fink, MD, PhD
Research Clinic for Functional Disorders and
Psychosomatics
Aarhus University Hospital
Nørrebrogade 44, bygn. 4, 1.
8000 Aarhus C

Lisbeth Frostholm, psychologist, PhD
Research Clinic for Functional Disorders and
Psychosomatics
Aarhus University Hospital
Nørrebrogade 44, bygn. 4, 1.
8000 Aarhus C

Allan 'Ben' Smith
Translational Research Fellow
Centre for Oncology Education and Research
Translation (CONCERT)
Ingham Institute for Applied Medical Research
1 Campbell Street Liverpool NSW 2170
Australia

Bo Andreassen Rix, MD, psychologist
Danish Cancer Society
Strandboulevarden 49
2100 København Ø

Table of Contents

BACKGROUND 4
AIM 5
METHODS/DESIGN 6
STUDY DESIGN 6
Survey 6
RCT 6
SETTING 6
STUDY POPULATION 7
Inclusion criteria for participating in the RCT: 7
Exclusion criteria for participating in the RCT: 7
PROCEDURE 8
Part 1, survey 8
Part 2, recruitment 8
Part 2 b, randomization 9
Part 3, pilot study 10
Part 4, intervention 10
Process of participation 11
EVALUATION 12
PRIMARY OUTCOME MEASURE 12
SECONDARY OUTCOME MEASURES 12
PROCESS MEASURES 13
REGISTER DATA 13
Baseline data 13
Economic measures 13
STATISTICAL PLAN 14
POWER CALCULATION 14
STATISTICAL ANALYSIS 14
Dropout analysis 14
Predictor analysis 14
Mediator analysis 15
Economic analysis 15
DATA MANAGEMENT 15
ETHICS 15
REASEARCH PLAN 16
DISSEMINATION 17
PLANNED ARTICLES: 17
DISCUSSION 17
PERSPECTIVES 17
REFERENCES 19

BACKGROUND

Colorectal cancer (CRC) screening, early detection and improved treatment have led to rising survival rates over the past decades. This improvement has resulted in an increasing number of long-term CRC survivors with no residual disease. Most survivors manage to establish a 'new normal' after finishing treatment, but some survivors experience difficulties in normal functioning and decreased quality of life (QoL) due to substantial psychological strain. One of the most common concerns among cancer survivors is fear of cancer recurrence (FCR)[1], defined as "*Fear, worry or concern relating to the possibility that cancer will come back or progress[2]*". The severity of self-reported FCR does not seem to differ much between different cancer types[3] and FCR can persist among very long term survivors[4]. Higher FCR is associated with several psychological factors including (health) anxiety[5], depression[6], perceived uncertainty in illness, perceived risk of recurrence and negative beliefs about worry[7, 8]. Clinically significant FCR (i.e. clinical FCR) is thought to be characterized by "*A preoccupation with the cancer returning or progressing, unhelpful coping behaviors, impairment to daily function, clinically significant distress and limited capacity for making future plans[2]*".

Most CRC survivors report some degree of FCR [9-12]. Two recent studies report likely clinically significant FCR among 13,7 % [13] and 10,1 % [14]of CRC survivors (unpublished data, personal communication) based on a validated measurement questionnaire (Fear of Cancer Recurrence Inventory, FCRI). Despite this the prevalence of clinical FCR in CRC survivors is still somewhat uncertain, as estimates are based on studies with small samples [n=51-91][10, 11, 13, 14], or studies with simple[9, 12] or unvalidated[11] FCR measures.
Two large cohort studies focusing on patient reported health-related quality of life after (colorectal) cancer do exists. The English study [15-17] includes people "living with and beyond cancer", which does not distinguish between survivors with no residual disease, with cancer still present, or with a history of recurrence and further more FCR is assessed by a single item. The Dutch PROFILES registry [3] includes no proposed cut-off score for clinical FCR.

A diagnosis of cancer is life changing and imposes heavy stress on patient and relatives. Together with often numerous physical symptoms and social changes after the cancer treatment, the net sum of stressors may exceed the cancer survivor's ability to adapt. This overload may manifest as a functional disorder/somatic symptom disorder such as bodily distress syndrome or health anxiety, as proposed by Simonelli et al [18]. Bodily distress syndrome is defined as a condition in which the patient suffers from, usually multiple, bodily symptoms in a characteristic symptom and illness pattern that are not attributable to verifiable, conventionally defined diseases [19].
Health anxiety is characterized by preoccupation with fear of having a serious and life-threatening illness with no objective sign of disease, and preoccupation persists despite medical reassurance [20]. Health anxiety and FCR obviously overlaps, as they also both includes unpleasant thoughts or ruminations, which interfere with everyday life and may lead to further unnecessary investigations and treatment. One study investigated hypochondriasis (the cousin of health anxiety) in breast cancer survivors and found that 43 % of those with clinical level of FCR met the diagnostic criteria [21]. Two studies of CRC patients have measured somatization, but not FCR [22, 23]. To the best of our knowledge, no previous studies have investigated the role of functional disorders in relation to FCR, anxiety and depression in CRC survivors.

Illness uncertainty has been linked with FCR [7] and health anxiety [24]. Illness uncertainty might by even greater when diagnosed asymptomatic as a consequence of screening. We postulate that screened to diagnosis leads to increased issues in coping with the cancer and the fear of recurrence. The comprehensive Danish Clinical Registries contain data on the method of diagnosis, e.g. if the CRC survivor is identified through the Danish Nation-wide Colorectal Cancer Screening Program, as opposed to a survivor initially diagnosed as a result of symptoms. This enables research in this unexplored area of psychosocial consequences of screen-detected cancers.

Around one fourth (26,5 %) of CRC survivors[16] and 20-56 % of people living with and beyond CRC cancer[25] report psychosocial assistance in coping with FCR to be an important unmet need. Randomized controlled trials testing interventions for reducing FCR have primarily been conducted in breast cancer survivors or mixed cancer populations. Most interventions are based on variations of cognitive-behavioral therapy (CBT). The delivery format of interventions previously or currently being evaluated has been in groups[26-28], face-to-face[29-31], blended[32], by telephone[33, 34] or by web-based platforms[35-37].
"ConquerFear" [29] is an individual face-to-face therapist-delivered intervention with demonstrated efficacy in reducing FCR versus a relaxation training attention control group in a mixed cancer sample of whom the majority (89%) were women with breast cancer. As this is a resource- and time-consuming approach accessible primarily to those in close proximity to major metropolitan cancer centres with highly trained psychologists, a web-based self-management version of ConquerFear has been created (iConquerFear), similar in curriculum content, but different in delivery.

Web-based interventions have the potential to fill an important gap in quality cancer care by augmenting limited available mental health services[38]. Web-based therapist-guided cognitive therapy has advantages for both patients and providers and effects appear comparable to traditional face-to-face therapy in treating distress in patients with cancer[39, 40]. Evidence suggests that guided interventions may be superior to unguided interventions [41].

AIM

The primary aim of this randomized controlled trial (RCT) is to test if a therapist-guided version of iConquerFear (TG-iConquerFear) can reduce FCR and improve QoL for CRC survivors more than augmented treatment as usual (augmented TAU).
Secondary objectives are to i) outline the prevalence of FCR in a population based CRC cohort up to 5 years post-diagnosis using a validated FCR measure with a clinical cut-off. This comprehensive screening will be used as recruitment for the RCT; ii) Investigate whether being diagnosed as a consequence of the Danish Nation-wide Colorectal Cancer Screening Program increases FCR compared to being diagnosed as a consequence of physical symptoms; and iii) investigate whether FCR is associated with anxiety, depression, bodily distress syndrome and health anxiety in CRC survivors as well as investigate whether uncertainty in illness, negative beliefs about worry and perceived risk of cancer recurrence act as moderators or mediators of FCR.

METHODS/DESIGN

Study design

Survey

This study is a population based cross sectional study. Participants will be invited to answer an electronic questionnaire to screen for fear of cancer recurrence and other psychological factors. The survey will be send out between September 2020 and September 2022.

RCT

This study is a population based randomized controlled clinical superiority trial. Participants are randomized to internet-based, TG-iConquerFear or augmented TAU (1:1), see Fig. 1. All participants will follow the standard cancer follow-up program.

Fig. 1, Trial design

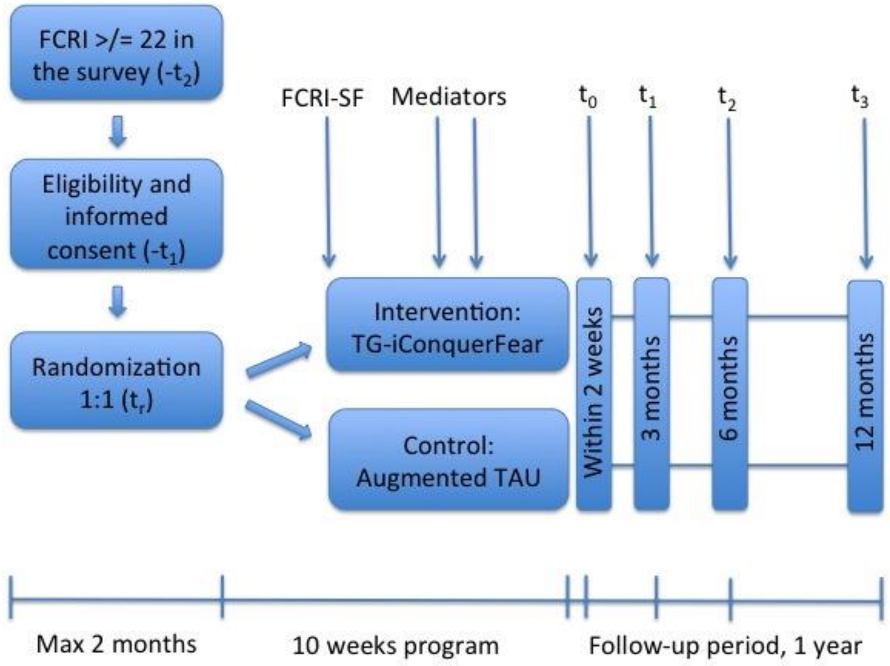

Setting

The idea of prioritizing psychosocial aspects of surviving cancer originates from patient and public involvement via the Patient's and Relative's Council at Lillebaelt Hospital and lead to the Clinic of Late Adverse Effect hosted by the Department of Oncology from where this project arises.

The supervisor team is composed based on qualification as HJ contribute with clinical cancer experience, LF with experience in ehealth and health anxiety, PF with massive knowledge in functional disorders and surveys and BS as an expert in FCR and the iConquerFear, as he participated in the development of the platform. As an extremely qualified representative from the largest patient organization in the country, BR contributes with non-clinical knowledge on cancer patients and a nationwide network to advertise for the project and disseminate results.

Experienced therapists working at the Center for Functional Disorders at Aarhus University Hospital will facilitate TG-iConquerFear. Sufficient introduction and training in iConquerFear will be ensured and supervision will be offered throughout the study. The primary investigator will be educated to perform telephone interview with study participants.
The self-directed version of iConquerFear was developed, adjusted and refined in Australia and then translated and adapted to a Danish context inspired by the cross-cultural adaptation process proposed by Beaton et al[42] with random samples translated forward-backward. An expert panel of therapists with expertise in internet-based treatment will perform further testing and adjusting on the raw material. A panel of volunteer, independent users from the Patient's and Relative's Council have continued the collaboration and will be invited to comment on all aspects of the study, including the platform and written material, and continue as an advisory board throughout the project period. Final testing will be done during the pilot study, see part 3.

Study population

All Danish CRC survivors above age 18 diagnosed after implementation of the CRC screening programme in March 2014 who have completed curative-intent cancer treatment with surgery and/or radiation and/or adjuvant chemotherapy will be invited to participate in the survey. Estimated number is 15,000 as the incidence is 5,000, and 80 % are offered curative intend treatment. One-year survival rate (data from 2011-2015) is 85 %. 5-year survival rate is 65 %. Overall survival rate used for this estimation is 75 %.
The RCT participants are recruited among the 15,000 colorectal cancer survivors invited to take part in survey. Through cooperation with the Danish Cancer Society we expect a slightly higher response rate than previous reported [43] of 60 %.
Among those 9,000, 12 % (1,080) are expected to score 22 or above on the FCRI-SF [13]. Approximately half of those are expected to be willing to participate in a randomised trial[37], resulting in 540 eligible participants.
The survivors will be identified through the Danish Colorectal Cancer Group (DCCG) database hosted by The Danish Clinical Registries (RKKP) with no need to enter individual patient records. The registry does not contain data on recurrence, and the cover letter will need to explain carefully, that in case of disease recurrence, the participants are asked to notify us in the first question or simply ignore the inquiry from us (see appendix 2. Følgebrev).

Inclusion criteria for participating in the RCT:

- Completed curative-intent cancer colorectal treatment with surgery and/or radiation and/or adjuvant chemotherapy between 01.03.14 and 31.12.18
- No history of recurrence after primary operation
- Fear of Cancer Recurrence Inventory score at or above 22
- Age above 18
- Read and understand Danish
- Access and ability to use Internet

Exclusion criteria for participating in the RCT:

- Severe psychiatric or cognitive disorder identified during telephone interview

- Cancer recurrence at any follow-up
- Substance abuse

Procedure

Part 1, survey

The CRC survivors will be invited by mail to their secure, personal electronic box (E-boks) to electronically fill out the 63-item screening questionnaire comprising: The FCRI-short form (FCRI-SF) assessing FCR severity, bodily distress symptoms (BDS checklist), health anxiety (Whiteley-8), anxiety (SCL4-anx), depression (SCL6-dep), and two items assessing global overall health and global quality of life (Visual Analogue Scale (VAS) 0-100). Two items asks for time since last cancer surveillance visit at the hospital and time until next visit. Participants are asked to answer yes or no regarding received chemotherapy or radiation. One item asks for consent to answers being used for research in anonymous form. Questionnaires and supplemental questions are listed in appendix 6 (Spørgeskemaer).
Completed questionnaires will be exported directly into the RedCap database for clinical research. Those with no access to Internet will be invited by paper and the data will be manually entered in RedCap. In case of no response, a reminder will be send after 2 weeks. All participants will be informed of their current level of FCR based on their answers.

Part 2, recruitment

In cases where participants in Part 1 report a FCRI-SF score ≥22 indicating likely clinically significant FCR [44], they will automatically be asked if they want to hear more about an experimental intervention targeting FCR, and 12 more questions appears on uncertainty in illness, negative beliefs about worry and perceived risk of cancer recurrence along with the full version of the FCRI (appendix 6). See Fig. 2 for timeline[45].

All survivors with likely clinically significant FCR who agree to be contacted by a research assistant (primary investigator or one of the two psychologists who will perform the intervention. All has been sufficient trained) will be telephoned, informed orally about the RCT and interviewed using the mini-SCAN, a brief semi-structured interview for psychiatric diagnosis [46]. This interview is designed to ensure appropriate treatment is offered to prospective study participants. If information and miniSCAN cannot be performed at the same time, a new appointment will be made. In case of severe depression or severe psychiatric or cognitive disorder, the survivor will be excluded from the study and encouraged to seek relevant help through their general practitioner. When needed, the research assistant can consult a psychiatrist. Supervision will be performed on regular basis.
The contacting research assistant will declare oral information given (appendix 4. Erklæring om videregivelse af mundtlig information) and for potential participants meeting eligibility criteria initiate for RedCap to send out written participant information sheet (appendix 3. Deltagerinformation) and consent form (appendix 5. Samtykkeerklæring til projektdeltagelse) to the participant's E-boks. By entering their Nem-id (a Danish secure personal coding system for electronic movements) they will consent to participation and concealed, computerized variable block randomization (1:1) stratified for age and gender will happen immediately.

Fig. 2, Timeline

	STUDY PERIOD					
	Population Screening	Enrolment	Randomization	ntervention	Follow-up	Close-out
**TIMEPOINT**	$-t_2$	$-t_1$	t_r	t_i	t_0, t_1, t_2	T_3
**ENROLMENT**		X				
**Screening**	X					
**Semistructured interview**		X				
**Informed consent**	X	X				
**Randomization**			X			
**INTERVENTIONS**						
TG-iConquerFear				X		
Augmented TAU				X		
**ASSESSMENTS**						
FCRI-SF	X			X		
Recurrence	X					
Supplementary	X					
Full FCRI		X			X	X
SCLs	X				X	X
BDS	X				X	X
Whiteley-8	X				X	X
QoL	X				X	X
MUIS		X		X + X	X	X
MCQ-30		X		X + X	X	X
Perceived risk		X		X + X	X	X

Part 2 b, randomization

If randomized to augmented TAU: The control group is described as “augmented” as the diagnostic telephone interview exceeds standard treatment. Furthermore, if randomized to augmented TAU the information letter (appendix 7. Informationsbrev) send to E-boks will include a reference to a website with a non-guided, publicly available E-learning program in cancer rehabilitation hosted by the Region of Central Jutland (livogkraeft.rm.dk). The website includes, besides a written material, self-help instructions for meditation. Use of the website will not be monitored.

If randomized to TG-iConquerFear: The information letter (Appendix 7. Informationsbrev) will contain a link to enter the platform directly. The assigned therapist will be informed simultaneously.
Both groups will follow standard surveillance according to national guidelines for cancer recurrence at a surgical or oncological department, which may includes colonoscopy and/or CT scans. The municipality is responsible for rehabilitation and offers limited access to physical exercise, yoga, dieticians, psychologists, sexologists etc.

Part 3, pilot study

The first 20 CRC survivors will be evaluated separately as a pilot group to explore barriers and facilitators for entering and completing internet-based therapy. Furthermore, we will optimize the feasibility of TG-iConquerFear in a small population before launching a comprehensive RCT. To ensure continuity between screening, recruitment, and the RCT no questionnaires will be send out while we evaluate the pilot study. If the pilot study does not raise any need for major changes in the study design or procedures, the pilot study will seamlessly proceed to the RCT.

Part 4, intervention

The theoretical frame of iConquerFear[29] is based on the Common-Sense Model (CSM) of illness, the Self-Regulatory Executive Function model (S-REF; targets metacognitions) and Relational Frame Theory (RFT; theoretical basis for Acceptance and Commitment Therapy). The intervention includes elements of attention training, increasing metacognitive awareness, acceptance & mindfulness, promotion of expedient screening behaviour, and values-based goal setting. The platform is accessed through secure log-on and comprises 5 modules containing educational text, interactive exercises, short videos featuring doctors, therapists and patients' perspectives, see Fig. 3.

The Australian version of iConquerFear is completely self-directed.
The Danish version of iConquerFear will contain a messenger-function on the intervention dashboard, allowing the participant and therapist to communicate asynchronously (TG-iConquerFear). The participant is guided through the sessions by minimum weekly contact with an experienced therapist. The therapist will motivate, answer questions and give feedback on written material and exercises. The amount of contact will be recorded and explored as a moderator of intervention efficacy. Should any cancer-related questions occur, an oncologist or surgeon will be involved as a consultant to the therapists.
The programme can monitor adherence, dropouts and track log-ins and activity. A purpose-developed single-item questionnaire within the TG-iConquerFear will each week ask for level of engagement[47]. Participants who do not adhere to, or withdraw from, the intervention will be contacted by telephone and asked why. These participants will be asked if they would be willing to complete a post-intervention assessment and all follow-up assessments to aid intervention evaluation. Therapist assistance is scheduled to last 10 weeks, but the participants will have free access to the platform for another 6 months.
In case of recurrence during the 10 weeks of intervention, the participant will be excluded from analysis, but may continue in the programme.

Fig. 3, iConquerFear curriculum content

Module	Content and Features
1. Introduction and orientation	• Introduction to FCR (survivor videos)<br>• Overview of FCR treatment model (interactive animation and therapist video)<br>• Values clarification (interactive card sort exercise)
2. Attention training	• Introduction to Attention Training (Interactive, therapist video, Attention Training audio, monitoring and feedback, regular practice reminders)
3. Detached mindfulness	• Introduction to Detached Mindfulness (Therapist videos)<br>• Demonstration of Detached Mindfulness exercises (Animated videos, therapist feedback)
4. Learning to live well and manage worry	• Psycho-education regarding appropriate threat monitoring behaviours (Annotated PowerPoint video)<br>• Assessing compliance with follow-up & self-examination recommendations (Interactive exercise with personalised feedback)<br>• Worry management techniques (Textual overview and downloadable PDF)
5. Treatment summery and relapse prevention	• Assessment and feedback on change in FCR symptoms during treatment (therapist feedback)<br>• Consolidation of newly acquired strategies for managing FCR through relapse prevention (therapist feedback and downloadable action plan)
Features common across all modules include: Write to your therapist, interactive exercises, downloadable handouts, progress graphs and safety plan.	

The design of the RCT and the procedure of delivering oral and written information and consenting by Nem-id is inspired by and has proven feasible in the protocol "A randomised controlled trial of Internet-based acceptance and commitment therapy (iACT) to patients with health anxiety" approved by the Regional Committee on Health Research Ethics for Central Jutland the 23rd November 2015, reference number ESDH 1-10-72-321-15. The process of participation is further elaborated below:

Process of participation

1. Receives "Følgebrev" and a survey (Spørgeskemaer, bilag 1 – screening) in E-boks.
2. Answers. Or reminded after 2 weeks
3. In case of FCRI under 22: No further participation
4. In case of FCRI at or above 22:
   - Informed of elevated level directly in the questionnaire
   - Asked if interested in hearing more about the RCT targeting FCR
   - Asked to enter phone number
   - Asked to fill out another questionnaire (45 items)
5. Telephoned by a research assistant, oral information about RCT is given (purpose, risks, benefits, voluntariness). Rescheduling in case of needing assessor.

6. Semistructured interview is performed by telephone. If possible at the same time as oral information is given, otherwise rescheduled.
7. In case of eligibility and wish to participate: Written information about RCT "Deltagerinformation", consent form "Samtykkeerklæring til projektdeltagelse" and "Før du beslutter dig"-pamphlet is send to E-boks
8. Consenting with Nem-id. Or reminded after 2 weeks of consideration.
9. No answer in 4 weeks is interpreted as not wanting to participate.
10. If consent is given: Randomization
11. Information letter "Informationsbrev" to E-boks, either with link to livogkraeft.rm.dk (control arm) or link to TG-iConquerFear (intervention arm)
12. Control arm or intervention arm for 10 weeks (Spørgeskemaer, bilag 3 – mediationsparametre)
13. 12 weeks after consent form is signed: 1st follow-up (Spørgeskemaer, bilag 4 – follow-up)
14. 12 weeks after 1st follow-up: 2nd follow-up
15. 12 weeks after 2nd follow-up: 3rd follow-up
16. 24 weeks after 3rd follow-up (One year after consent form is signed): 4th and final follow-up

EVALUATION

Primary outcome measure

The primary outcome measure is the 3 month follow-up (T1) total score on the recently validated Danish version of the FCRI [13]. The FCRI is a 42-item self-report measure that includes subscales assessing FCR triggers, severity, psychological distress, functioning impairments, insight, reassurance, and coping strategies [48]. The FCRI has demonstrated high: internal consistency (α=0.96) [49], interclass correlation (0.84) [13] and convergent validity in large heterogeneous cancer survivor samples. Respondents rate the degree to which symptoms/issues affected them over the past month on a Likert scale ranging from 0 ('not at all' or 'never') to 4 ('a great deal' or 'all the time'). While there are potential issues with aggregating FCRI subscales [50], total FCRI score will be used as the dependent variable, as it closely reflects proposed features of clinically significant FCR; namely related distress, functional impact, and maladaptive coping[2], rather than the level of fear indicated by the FCRI-SF [50, 51]. Total scores can range from 0-168; higher scores indicate greater FCR morbidity. Level of FCR will be measured post-treatment (T0) and at follow-up after 3 (T1) and 6 (T2) months. Long-term effect will be evaluated after one year (T3). All electronic questionnaires will be sent out automatically through RedCAP.

Secondary outcome measures

Change from baseline (Tb) to post-intervention (T0, T1, T2, T3) in the following outcomes

- *Bodily Distress Symptom/somatization, Anxiety and depression* evaluated by relevant Symptom Checklist-90-R (SCL) subscales [52], validated in Danish by Olsen et al [53].
- *Health Anxiety* measured by the validated [54] Whiteley-8 index [55].
- *Global quality of life* and *global health* measured by a VAS 0-100

Process measures

Variables likely to mediate the impact of TG-iConquerFear according to the cognitive processing and blended theoretical models of FCR and the existing research will be measured twice during the intervention and at follow-up

- *Uncertainty in illness* measured by Mishels Uncertainty of Illness Scale (MUIS) [56], validated in a short form [57]. Translated into Danish during this study.
- *Negative beliefs about worry* is a validated subscale [58] of the MetaCognitions Questionnaire-30 [59] targeting metacognition. Translated into Danish in 2009 at Center for Psychiatric Research at Aarhus University Hospital.
- *Perceived risk of recurrence* is measured by a visual analogue scale from 1-100 as presented by Lebel et al [7]. Translated into Danish during this study.

Register data

Baseline data

From the Danish Clinical Registries (RKKP) the following variables will be obtained prior to the RCT:

- Age
- Sex
- Participation in the Danish Colorectal Cancer Screening Program (data from the Danish Screening Database). “Yes” is annotated for individuals who submit a stool sample within three months after receiving screening invitation.
- Cancer type (colon / rectum)
- TNM stage
- Localization (left side or right side)
- Date of diagnosis
- Date of surgery
- Type of surgery
- Surgery performed with curative intent
- Radical procedure (yes / no)
- In case of no surgery performed: Cause
- Stoma (no / temporary / yes)
- Performance status
- Charlson score

Economic measures

For evaluating cost-effectiveness of TG-iConquerFear and for comparison of changes in health care usage between intervention arm and augmented TAU, information from multiple Danish registries will be extracted. Data will be obtained at end of study for the retrospective period from intervention start to final follow-up, maximum 15 months.

From the Patient Registry (LPR) the following variables will be obtained:

- Hospitalization, duration and diagnosis
- Ambulatory visits, date

From the Health Insurance Registry (SSR) the following variable will be obtained:

- Total cost for use of primary care physician, physiotherapist and psychologist

From Drug Statistics Registry (LSR) the following variable will be obtained:

- Use of psychiatric related drugs (anti-depressive drugs, anxiolytics etc.)

From the DREAM database hosted by the ministry of employment, the following variables will be obtained:

- Sickness absence
- Leave of absence
- Marital status (for baseline)

STATISTICAL PLAN

Power calculation

The sample size is calculated for the primary outcome FCR measured by the FCRI. To detect a standardised mean difference in FCR (Cohen's d = 0.5, group difference of 3,5 and a standard deviation of 7) with 90 % power and two-sided alpha = 0,05 with to-sided t-test a sample size of 246 participants is desired. With a realistic dropout of 30 %, 350 participants are required in each group, and it seems realistic to recruit this as a minimum among the estimated number of eligible participants.

Statistical analysis

A flowchart of participants and dropouts following the CONSORT guidelines will be drawn. STATA will be used for all statistical analyses. All statistical tests will be two-side (level of significance = 0.05). χ^2-tests will be performed on all follow-up data to analyse proportions of participants above or below the clinical cut-off.
The influence of the intervention will be analysed for the primary outcome and for all secondary outcomes. Linear mixed effects models will be used to analyse longitudinal differences between intervention and control group. Association with functional disorders (health anxiety and bodily distress symptom) and psychiatric disorders (anxiety and depression) will be evaluated with adjusted and unadjusted linear regressions analyses on baseline data.

Dropout analysis

Available demographic data and psychosocial information will be compared and evaluated for participants and dropouts to assess generalizability. Any differences will be taken into account in later analyses.

Predictor analysis

The moderating effect of age, gender and cancer-related characteristics (stage of disease (I-IV), type of surgery, screening vs. non-screening e.g.) and actual situation (time since last visit at hospital, time until next visit, stoma) will be analysed using linear regressions analyses.

Mediator analysis

The mediators uncertainty in illness, perceived risk of recurrence and negative beliefs about worry will be analysed by regression models with possible interaction with the intervention.

Economic analysis

Multivariate analysis will be performed to explore any difference between the intervention and control group. The economic cost of the average participant in each group will be calculated based on register data.

DATA MANAGEMENT

The electronic data capture system RedCap will be used for data management with double data entry of paper questionnaires. Paper questionnaires will be shredded. During the study data will be processed and stored in accordance with applicable legislation (EU GDPR and the Danish Data Protection Act), using REDCap and OPEN Analyse via OPEN Odense Patient data Explorative Network, Odense University Hospital, Region of Southern Denmark.

The Research Clinic for Functional Disorders and Psychosomatics, Aarhus University Hospital has signed a data management agreement with Crystone A/B in Sweden. All personal information including name, phone number, social security number and data related to the intervention-platform are encrypted and stored on their server. The survey answers are stored at a random generated id.

When the study is finished, data will be transferred to the National Archives. Access to data is limited to listed authors by passwords. All data will be handled with confidentiality.

ETHICS

Permission will be obtained from The Regional Committees on Health Research Ethics for Southern Denmark. The project is notified to the Regional Register of Health Related Research. Patients will follow the standard surveillance of cancer recurrence. Enrolled patients will not be exposed to further examinations with blood sampling or radiation, so there is no risk of physical harm for the patients. Patients randomised to the control-group will still be handled according to current standards and not receive any less attention.

No research indicates side effects of Internet-based treatment to cancer survivors. The assigned psychologist will register and follow any serious adverse effects until the problem is solved, stabilized or association with the study has been dismissed. Serious adverse effects will be reported to sponsor in one working day. Sponsor will report to the Ethical Committee once a year.

The participants can at all time withdraw from the study without explanation.

Participants are covered by “Patienterstatningen”. No remuneration will be offered. The Helsinki declaration will be met.

The project may give new knowledge about improved care for cancer survivors and thus the benefits outweigh the potential harms.

REASEARCH PLAN

The protocol is part of a PhD project at the University of Southern Denmark planned from January 1st 2020 and the following 5 years parallel with specialist training, see Fig. 4 for elaboration.

Key dates:

- May 2020: Extract data from RKKP
- September 2020: Launch survey
- October 2020: Inclusion of first participant
- April 2022: RCT finish
- May 2023: Final 1-year follow-up
- January 2024: Extraction of retrospective economic data

Fig. 4, research and study plan

Research and study plan																									
	2018	2019				2020				2021				2022				2023				2024			
Activity	Q4	Q1	Q2	Q3	Q4	Q1	Q2	Q3	Q4	Q1	Q2	Q3	Q4	Q1	Q2	Q3	Q4	Q1	Q2	Q3	Q4	Q1	Q2	Q3	Q4
Preparation studies<br>- Protocol<br>- Ethics<br>- Review<br>- Fundraise<br>- REDCap																									
Specialist training																									
Platform work<br>- Translate<br>- Test																									
miniSCAN education & training																									
Launch survey (recruitment)																									
Perform miniSCAN (inclusion)																									
Platform work<br>- Pilot test																									
Launch RCT (intervention)																									
RCT runs																									
Status / Evaluation																									
Follow-up ½, 3, 6 & 12 months																									
Exchange in Australia																									
Analysing																									

Funding (VIP and TAP salary, statistical assistance, questionnaires, overhead e.g.) will be sought from public and private funds, and handled by the Department of Clinical Oncology, Vejle Hospital. The

Ph.D. student is a regular member of the Danish Cancer Society, but do not have any financial connection to any of the stakeholders.

DISSEMINATION

Results will be published in international, peer-reviewed journals that reach an appropriate audience (open access where possible). The RCT will be registered at www.clinicaltrials.gov with a reference to results. Data will be presented at national and international congresses. Negative, positive and inconclusive results will be published. An oral, public PhD defence will be held. If shown to be efficacious the results will be presented at cancer-treating departments throughout Denmark to encourage implementation. Results will be published at the Danish Cancer Society's webpage to inform future users. Study participants will if interested be mailed the results when the study is finished.

Planned articles:

1. Therapist guided iConquerFear Protocol: Randomised controlled trial of therapist guided internet-delivered cognitive therapy for fear of cancer recurrence in colorectal cancer survivors vs. augmented treatment as usual
2. Fear of cancer recurrence in colorectal cancer survivors: A Danish population based study
3. Fear of cancer recurrence in colorectal cancer survivors screened to diagnosis
4. Fear of cancer recurrence in colorectal cancer survivors and association to functional disorders
5. Therapist guided iConquerFear targeting fear of cancer recurrence in colorectal cancer survivors

DISCUSSION

This study is the first to analyse the effect of a therapeutic guided, Internet-based intervention for CRC survivors with clinical levels of FCR. The developmental process has included CRC survivors, oncology nurses, psychiatrists, psychologists, the Patient' and Relative's Council at Lillebaelt Hospital and doctors working in the field of oncology. Internet delivery was chosen, as many CRC survivors are working, and traditionally men are more reluctant to participate in face-to-face therapy. We believe this combination of a careful development and a considerate delivery will make TG-iConquerFear successful in reducing FCR and has the potential to target more men than face-to-face formats.

PERSPECTIVES

This study will add to the current but sparse knowledge on optimal treatment of FCR among colorectal cancer survivors. Data from this project can be used directly as guidance in clinical daily life. At the current time no guidelines exists.
If successful, TG-iCF is proved effective in reducing psychological morbidity among colorectal cancer survivors and overall health care costs due to more appropriate use of health care services. Not only colorectal cancer survivors experiences fear of recurrence of disease, and TG-iCF have the

potential to be extended to multiple different diagnoses, including other cancer diagnoses but also survivors of cardiac arrest, acute lung embolism, TCI, apoplexies, etc.

REFERENCES

1. Simard S, Thewes B, Humphris G, Dixon M, Hayden C, Mireskandari S, Ozakinci G: **Fear of cancer recurrence in adult cancer survivors: a systematic review of quantitative studies**. *Journal of cancer survivorship : research and practice* 2013, **7**(3):300-322.
2. Lebel S, Ozakinci G, Humphris G, Mutsaers B, Thewes B, Prins J, Dinkel A, Butow P, University of Ottawa Fear of Cancer Recurrence Colloquium a: **From normal response to clinical problem: definition and clinical features of fear of cancer recurrence**. *Supportive care in cancer : official journal of the Multinational Association of Supportive Care in Cancer* 2016, **24**(8):3265-3268.
3. van de Wal M, van de Poll-Franse L, Prins J, Gielissen M: **Does fear of cancer recurrence differ between cancer types? A study from the population-based PROFILES registry**. (1099-1611 (Electronic)).
4. Koch L, Jansen L Fau - Brenner H, Brenner H Fau - Arndt V, Arndt V: **Fear of recurrence and disease progression in long-term (>/= 5 years) cancer survivors--a systematic review of quantitative studies**. (1099-1611 (Electronic)).
5. McGinty HL, Small BJ, Laronga C, Jacobsen PB: **Predictors and patterns of fear of cancer recurrence in breast cancer survivors**. *Health psychology : official journal of the Division of Health Psychology, American Psychological Association* 2016, **35**(1):1-9.
6. Deimling GT, Bowman KF, Sterns S, Wagner LJ, Kahana B: **Cancer-related health worries and psychological distress among older adult, long-term cancer survivors**. *Psychooncology* 2006, **15**(4):306-320.
7. Lebel S, Maheu C, Tomei C, Bernstein LJ, Courbasson C, Ferguson S, Harris C, Jolicoeur L, Lefebvre M, Muraca L *et al*: **Towards the validation of a new, blended theoretical model of fear of cancer recurrence**. *Psychooncology* 2018, **27**(11):2594-2601.
8. Butow P, Kelly S, Thewes B, Hruby G, Sharpe L, Beith J: **Attentional bias and metacognitions in cancer survivors with high fear of cancer recurrence**. *Psychooncology* 2015, **24**(4):416-423.
9. Waters EA, Arora Nk Fau - Klein WMP, Klein Wm Fau - Han PKJ, Han PK: **Perceived risk, trust and health-related quality of life among cancer survivors**. (1532-4796 (Electronic)).
10. Mullens AB, McCaul Kd Fau - Erickson SC, Erickson Sc Fau - Sandgren AK, Sandgren AK: **Coping after cancer: risk perceptions, worry, and health behaviors among colorectal cancer survivors**. (1057-9249 (Print)).
11. Steele N, Haigh R Fau - Knowles G, Knowles G Fau - Mackean M, Mackean M: **Carcinoembryonic antigen (CEA) testing in colorectal cancer follow up: what do patients think?** (1469-0756 (Electronic)).
12. Fisher A, Beeken RJ, Heinrich M, Williams K, Wardle J: **Health behaviours and fear of cancer recurrence in 10 969 colorectal cancer (CRC) patients**. *Psychooncology* 2016, **25**(12):1434-1440.
13. Hovdenak Jakobsen I, Jeppesen MM, Simard S, Thaysen HV, Laurberg S, Juul T: **Initial validation of the Danish version of the Fear of Cancer Recurrence Inventory (FCRI) in colorectal cancer patients**. *J Cancer Surviv* 2018, **12**(6):723-732.
14. Custers JAE, Gielissen MFM, Janssen SHV, de Wilt JHW, Prins JB: **Fear of cancer recurrence in colorectal cancer survivors**. *Support Care Cancer* 2016, **24**(2):555-562.
15. Glaser AW, Fraser Lk Fau - Corner J, Corner J Fau - Feltbower R, Feltbower R Fau - Morris EJA, Morris Ej Fau - Hartwell G, Hartwell G Fau - Richards M, Richards M Fau - Wagland R, Wagland R: **Patient-reported outcomes of cancer survivors in England 1-5 years after diagnosis: a cross-sectional survey. LID - 10.1136/bmjopen-2012-002317 [doi] LID - e002317 [pii]**. (2044-6055 (Print)).
16. Jefford M, Ward AC, Lisy K, Lacey K, Emery JD, Glaser AW, Cross H, Krishnasamy M, McLachlan SA, Bishop J: **Patient-reported outcomes in cancer survivors: a population-wide cross-sectional study**. (1433-7339 (Electronic)).

17. Downing A, Morris EJ, Richards M, Corner J, Wright P, Sebag-Montefiore D, Finan P, Kind P, Wood C, Lawton S *et al*: **Health-related quality of life after colorectal cancer in England: a patient-reported outcomes study of individuals 12 to 36 months after diagnosis**. (1527-7755 (Electronic)).
18. Simonelli LE, Siegel SD, Duffy NM: **Fear of cancer recurrence: a theoretical review and its relevance for clinical presentation and management**. *Psychooncology* 2017, **26**(10):1444-1454.
19. Fink P, Schroder A: **One single diagnosis, bodily distress syndrome, succeeded to capture 10 diagnostic categories of functional somatic syndromes and somatoform disorders**. *Journal of psychosomatic research* 2010, **68**(5):415-426.
20. Tyrer P, Eilenberg T, Fink P, Hedman E, Tyrer H: **Health anxiety: the silent, disabling epidemic**. *BMJ (Clinical research ed)* 2016, **353**:i2250.
21. Thewes B, Bell ML, Butow P, Beith J, Boyle F, Friedlander M, McLachlan SA: **Psychological morbidity and stress but not social factors influence level of fear of cancer recurrence in young women with early breast cancer: results of a cross-sectional study**. *Psychooncology* 2013, **22**(12):2797-2806.
22. Chambers SK, Baade P, Meng X, Youl P, Aitken J, Dunn J: **Survivor identity after colorectal cancer: antecedents, prevalence and outcomes**. *Psychooncology* 2012, **21**(9):962-969.
23. Dunn J, Ng SK, Holland J, Aitken J, Youl P, Baade PD, Chambers SK: **Trajectories of psychological distress after colorectal cancer**. *Psychooncology* 2013, **22**(8):1759-1765.
24. Eilenberg T, Kronstrand L, Fink P, Frostholm L: **Acceptance and commitment group therapy for health anxiety--results from a pilot study**. *Journal of anxiety disorders* 2013, **27**(5):461-468.
25. Kotronoulas G, Papadopoulou C, Burns-Cunningham K, Simpson M, Maguire R: **A systematic review of the supportive care needs of people living with and beyond cancer of the colon and/or rectum**. *European journal of oncology nursing : the official journal of European Oncology Nursing Society* 2017, **29**:60-70.
26. Herschbach P, Book K, Dinkel A, Berg P, Waadt S, Duran G, Engst-Hastreiter U, Henrich G: **Evaluation of two group therapies to reduce fear of progression in cancer patients**. *Supportive care in cancer : official journal of the Multinational Association of Supportive Care in Cancer* 2010, **18**(4):471-479.
27. Maheu C, Lebel S, Courbasson C, Lefebvre M, Singh M, Bernstein LJ, Muraca L, Benea A, Jolicoeur L, Harris C *et al*: **Protocol of a randomized controlled trial of the fear of recurrence therapy (FORT) intervention for women with breast or gynecological cancer**. (1471-2407 (Electronic)).
28. Merckaert I, Lewis F, Delevallez F, Herman S, Caillier M, Delvaux N, Libert Y, Lienard A, Nogaret JM, Ogez D *et al*: **Improving anxiety regulation in patients with breast cancer at the beginning of the survivorship period: a randomized clinical trial comparing the benefits of single-component and multiple-component group interventions**. (1099-1611 (Electronic)).
29. Butow PN, Turner J, Gilchrist J, Sharpe L, Smith AB, Fardell JE, Tesson S, O'Connell R, Girgis A, Gebski VJ *et al*: **Randomized Trial of ConquerFear: A Novel, Theoretically Based Psychosocial Intervention for Fear of Cancer Recurrence**. *Journal of clinical oncology : official journal of the American Society of Clinical Oncology* 2017, **35**(36):4066-4077.
30. Tomei CA-Ohoo, Lebel S, Maheu C, Lefebvre M, Harris C: **Examining the preliminary efficacy of an intervention for fear of cancer recurrence in female cancer survivors: a randomized controlled clinical trial pilot study**. (1433-7339 (Electronic)).
31. Davidson J, Malloch M, Humphris GA-Ohoo: **A single-session intervention (the Mini-AFTERc) for fear of cancer recurrence: A feasibility study**. (1099-1611 (Electronic)).
32. van de Wal M, Thewes B, Gielissen M, Speckens A, Prins J: **Efficacy of Blended Cognitive Behavior Therapy for High Fear of Recurrence in Breast, Prostate, and Colorectal Cancer Survivors: The SWORD Study, a Randomized Controlled Trial**. *Journal of clinical oncology : official journal of the American Society of Clinical Oncology* 2017, **35**(19):2173-2183.

33. Cruickshank S, Steel E, Fenlon D, Armes J, Scanlon K, Banks E, Humphris G: **A feasibility study of the Mini-AFTER telephone intervention for the management of fear of recurrence in breast cancer survivors: a mixed-methods study protocol**. (2055-5784 (Print)).
34. Dieng M, Butow PN, Costa DS, Morton RL, Menzies SW, Mireskandari S, Tesson S, Mann GJ, Cust AE, Kasparian NA: **Psychoeducational Intervention to Reduce Fear of Cancer Recurrence in People at High Risk of Developing Another Primary Melanoma: Results of a Randomized Controlled Trial**. *Journal of clinical oncology : official journal of the American Society of Clinical Oncology* 2016, **34**(36):4405-4414.
35. van Helmondt SJ, van der Lee ML, de Vries J: **Study protocol of the CAREST-trial: a randomised controlled trial on the (cost-) effectiveness of a CBT-based online self-help training for fear of cancer recurrence in women with curatively treated breast cancer**. *BMC cancer* 2016, **16**:527-016-2562-2560.
36. Murphy MJ, Newby JM, Butow P, Kirsten L, Allison K, Loughnan S, Price MA, Shaw J, Shepherd H, Smith J *et al*: **iCanADAPT Early protocol: randomised controlled trial (RCT) of clinician supervised transdiagnostic internet-delivered cognitive behaviour therapy (iCBT) for depression and/or anxiety in early stage cancer survivors -vs- treatment as usual**. (1471-2407 (Electronic)).
37. Willems RA, Mesters I, Lechner L, Kanera IM, Bolman CAW: **Long-term effectiveness and moderators of a web-based tailored intervention for cancer survivors on social and emotional functioning, depression, and fatigue: randomized controlled trial**. *J Cancer Surviv* 2017, **11**(6):691-703.
38. Leykin Y, Thekdi SM, Shumay DM, Munoz RF, Riba M, Dunn LB: **Internet interventions for improving psychological well-being in psycho-oncology: review and recommendations**. *Psychooncology* 2012, **21**(9):1016-1025.
39. Compen F, Bisseling E, Schellekens M, Donders R, Carlson L, van der Lee M, Speckens A: **Face-to-Face and Internet-Based Mindfulness-Based Cognitive Therapy Compared With Treatment as Usual in Reducing Psychological Distress in Patients With Cancer: A Multicenter Randomized Controlled Trial**. *Journal of clinical oncology : official journal of the American Society of Clinical Oncology* 2018, **36**(23):2413-2421.
40. Spek V, Cuijpers P, Nyklicek I, Riper H, Keyzer J, Pop V: **Internet-based cognitive behaviour therapy for symptoms of depression and anxiety: a meta-analysis**. *Psychological medicine* 2007, **37**(3):319-328.
41. Pihlaja S, Stenberg JH, Joutsenniemi K, Mehik H, Ritola V, Joffe G: **Therapeutic alliance in guided internet therapy programs for depression and anxiety disorders - A systematic review**. *Internet interventions* 2018, **11**:1-10.
42. Beaton DE, Bombardier C, Guillemin F, Ferraz MB: **Guidelines for the process of cross-cultural adaptation of self-report measures**. *Spine* 2000, **25**(24):3186-3191.
43. McMullen C, Bulkley J, Corley DA, Madrid S, Davis AQ, Hesselbrock R, Kurtilla F, Anderson CK, Arterburn D, Somkin CP *et al*: **Health care improvement and survivorship priorities of colorectal cancer survivors: findings from the PORTAL colorectal cancer cohort survey**. *Support Care Cancer* 2019, **27**(1):147-156.
44. Fardell JE, Jones G, Smith AB, Lebel S, Thewes B, Costa D, Tiller K, Simard S, Feldstain A, Beattie S *et al*: **Exploring the screening capacity of the Fear of Cancer Recurrence Inventory-Short Form for clinical levels of fear of cancer recurrence**. *Psycho-oncology* 2018, **27**(2):492-499.
45. Chan AW, Tetzlaff JM, Gotzsche PC, Altman DG, Mann H, Berlin JA, Dickersin K, Hrobjartsson A, Schulz KF, Parulekar WR *et al*: **SPIRIT 2013 explanation and elaboration: guidance for protocols of clinical trials**. *BMJ (Clinical research ed)* 2013, **346**:e7586.
46. Nienhuis FJ, van de Willige G, Rijnders CA, de Jonge P, Wiersma D: **Validity of a short clinical interview for psychiatric diagnosis: the mini-SCAN**. *The British journal of psychiatry : the journal of mental science* 2010, **196**(1):64-68.

47. Short CE, DeSmet A, Woods C, Williams SL, Maher C, Middelweerd A, Muller AM, Wark PA, Vandelanotte C, Poppe L *et al*: **Measuring Engagement in eHealth and mHealth Behavior Change Interventions: Viewpoint of Methodologies**. *Journal of medical Internet research* 2018, **20**(11):e292.
48. Simard S, Savard J: **Fear of Cancer Recurrence Inventory: Development and initial validation of a multidimensional measure of fear of cancer recurrence**. *Support Care Cancer* 2009, **17**(3):241-251.
49. Lebel S, Simard S, Harris C, Feldstain A, Beattie S, McCallum M, Lefebvre M, Savard J, Devins GM: **Empirical validation of the English version of the Fear of Cancer Recurrence Inventory**. *Quality of life research : an international journal of quality of life aspects of treatment, care and rehabilitation* 2016, **25**(2):311-321.
50. Costa DSJ, Smith AB, Fardell JE: **The sum of all fears: conceptual challenges with measuring fear of cancer recurrence**. *Support Care Cancer* 2016, **24**(1):1-3.
51. Costa DSJ: **Screening for clinical levels of fear of cancer recurrence**. *Psychooncology* 2017.
52. Parloff MB, Kelman HC, Frank JD: **Comfort, effectiveness, and self-awareness as criteria of improvement in psychotherapy**. *The American journal of psychiatry* 1954, **111**(5):343-352.
53. Olsen LR, Mortensen EL, Bech P: **The SCL-90 and SCL-90R versions validated by item response models in a Danish community sample**. *Acta psychiatrica Scandinavica* 2004, **110**(3):225-229.
54. Fink P, Ewald H, Jensen J, Sorensen L, Engberg M, Holm M, Munk-Jorgensen P: **Screening for somatization and hypochondriasis in primary care and neurological in-patients: a seven-item scale for hypochondriasis and somatization**. *Journal of psychosomatic research* 1999, **46**(3):261-273.
55. Pilowsky I: **Dimensions of hypochondriasis**. *The British journal of psychiatry : the journal of mental science* 1967, **113**(494):89-93.
56. Mishel MH: **The measurement of uncertainty in illness**. *Nursing research* 1981, **30**(5):258-263.
57. Hagen KB, Aas T, Lode K, Gjerde J, Lien E, Kvaloy JT, Lash TL, Soiland H, Lind R: **Illness uncertainty in breast cancer patients: validation of the 5-item short form of the Mishel Uncertainty in Illness Scale**. *European journal of oncology nursing : the official journal of European Oncology Nursing Society* 2015, **19**(2):113-119.
58. Wells A, Cartwright-Hatton S: **A short form of the metacognitions questionnaire: properties of the MCQ-30**. *Behaviour research and therapy* 2004, **42**(4):385-396.
59. Cartwright-Hatton S, Wells A: **Beliefs about worry and intrusions: the Meta-Cognitions Questionnaire and its correlates**. *Journal of anxiety disorders* 1997, **11**(3):279-296.

Version: 1.0
Date: 25 September 2020
Trial: TG-iConquerFear – Randomized Controlled Trial
SAP author: Johanne Dam Lyhne, MD, PhD and Signe Timm (statistician)

1. Background and objectives

This SAP pertains to the randomized controlled trial evaluating the therapist-guided eHealth intervention TG-iConquerFear versus an augmented self-help control for reducing fear of cancer recurrence (FCR) in colorectal cancer survivors.

Primary hypothesis

- TG-iConquerFear will result in a greater reduction in FCRI total score at 3 months post-intervention compared with control.

Secondary hypotheses

- TG-iConquerFear will reduce anxiety, depression, and distress, and improve quality of life.
- TG-iConquerFear will not affect the prevalence of physical symptoms.
- Some participants will demonstrate particularly high engagement and adherence with the online format, which may be associated with distinct demographic or psychosocial characteristics.

2. Sample size

- **Initial a priori calculation:** 350 participants required to detect a standardized mean difference of Cohen's *d* = 0.50 (mean difference = 3.5; SD = 7), with 90% power, 2-sided α = 0.05, and 30% dropout.

3. Analysis populations

- **Intention-to-treat (ITT):** All randomized participants analyzed according to their assigned group, regardless of adherence. The ITT population will form the primary analysis set.
- **Per-protocol (PP):** All participants who adhere to the intervention (>2 of 6 modules completed in TG-iConquerFear, or equivalent engagement with control condition). PP analyses will be performed to examine the robustness of ITT findings.
- **Exploratory subgroups:** Completers (6 modules) versus partial completers (2–5 modules). Analyses will also explore differences in demographic and psychosocial characteristics between subgroups.

4. Outcomes

Primary outcome

- Change in FCRI total score from baseline to 3 months.

Secondary outcomes

- Anxiety, depression, and distress (validated scales).

- Health-related quality of life (EQ-5D-5L).
- Proportion of participants scoring above/below the FCRI-SF cut-off (≥22).
- Adherence and engagement metrics (number of modules completed, communication with therapist).

Exploratory outcomes

- Dropout patterns (demographic and psychosocial predictors of attrition).
- Process measures (eg, illness uncertainty, perceived risk of recurrence, negative beliefs about worry) as potential mediators.
- Associations with functional somatic and psychiatric disorders (using validated cut-offs).

5. Statistical methods

5.1 Descriptive statistics

- Baseline demographics and cancer-related characteristics summarized as means (SD), medians (IQR), or proportions.
- Between-group balance assessed using standardized differences (>0.20 = imbalance).

5.2 Primary analysis

- Within-group changes from baseline to follow-up assessed with paired *t*-tests.
- Between-group differences in mean change scores tested using 2-sample *t*-tests (or non-parametric equivalent if assumptions violated).
- Effect size (Cohen's *d*) and 95% CI reported for between-group differences at 3 months.

5.3 Secondary analyses

- Continuous secondary outcomes analyzed using paired *t*-tests and between-group comparisons of change scores.
- Longitudinal analyses across baseline, ½, 3, and 6 months using **linear mixed-effects models**, with fixed effects for group, time, and interaction, and random intercepts for subjects.
- Proportions above/below clinical cut-offs analyzed using **χ^2 tests**.
- Reliable and clinically significant change indices calculated.

5.4 Per-protocol analyses

- Primary and secondary outcomes re-analyzed in the PP population to assess consistency with ITT results.

5.5 Exploratory analyses

- **Adherence/engagement:** descriptive statistics of modules completed, skew assessed, medians and IQR reported.
- **Dropout analysis:** logistic regression comparing demographic and psychosocial predictors of dropout vs retention.
- **Process measures:** regression models to examine mediators (illness uncertainty, perceived risk, negative beliefs about worry).
- **Associations with functional disorders:** logistic regression for dichotomized outcomes (eg, anxiety, depression, bodily distress) using established cut-offs.

6. Missing data

- Missing items on baseline patient-reported measures replaced with **0** (conservative assumption).
- If >50% of items missing for a scale, no score calculated.
- No imputation planned.

7. Multiplicity

- No formal adjustments planned. Secondary and exploratory analyses considered hypothesis-generating.

8. Significance level and software

- 2-sided $P < .05$ considered statistically significant.
- Analyses performed in **Stata 18** (StataCorp, College Station, TX).

Update September 2023

Updated calculation: Based on published ConquerFear group trial data, 68 participants would be sufficient to detect a significant difference with 95% power and 20% dropout.
Reference: *ConquerFear-Group: A randomized controlled trial of an online-delivered group-based psychological intervention for fear of cancer recurrence in breast cancer survivors* Tauber et al, Psychooncology, Sep 2023.

Planned decision: The revised sample size was adjusted to reflect the larger expected effect size from prior data.

Update 10 Jan 2025

Deviations from SAP

Several analyses specified in the statistical analysis plan were not conducted due to limitations in sample size and data availability.

- Per-protocol analyses were not performed, as the five participants classified as passive users (<2 modules completed) did not contribute outcome data, precluding meaningful subgroup comparison.
- Mediator analyses (eg, illness uncertainty, perceived risk of recurrence, negative beliefs about worry) were not conducted because of insufficient statistical power.
- Predictor analyses of demographic and clinical moderators (eg, age, gender, disease stage, treatment type) were omitted for the same reason.
- Economic analyses, originally planned using registry data, were not undertaken due to lack of power and feasibility constraints.
