# Supplementary material for: E-Health Intervention for Fear of Cancer Recurrence: A Randomized Clinical Trial
Source: JAMA Netw Open. 2025 Nov 11;8(11):e2542112. doi: 10.1001/jamanetworkopen.2025.42112 (PMC12606383; doi:10.1001/jamanetworkopen.2025.42112)
Supplement: Supplement 2. — eTable 1. Change in FCRI Triggers Subscale Between Baseline (T0) and Postintervention (T1) and 3- and 6-Month Follow-Up (T2 and T3) eTable 2. Change in FCRI Psychological Distress Subscale Between Baseline (T0) and Postintervention (T1) and 3- and 6-Month Follow-Up (T2 and T3) eTable 3. Change in FCRI Functional Impairment Subscale Between Baseline (T0) and Postintervention (T1) and 3- and 6-Month Follow-Up (T2 and T3) eTable 4. Change in FCRI Insight Subscale Between Baseline (T0) and Postintervention (T1) and 3- and 6-Month Follow-Up (T2 and T3) eTable 5. Change in FCRI Reassurance Subscale Between Baseline (T0) and Postintervention (T1) and 3- and 6-Month Follow-Up (T2 and T3) eTable 6. Change in FCRI Coping Strategies Subscale Between Baseline (T0) and Postintervention (T1) and 3- and 6-Month Follow-Up (T2 and T3) eTable 7. Process Measures: Change in Outcome During Intervention Compared to Baseline eTable 8. Characteristics of Completers Compared to Noncompleters [file jamanetwopen-e2542112-s002.pdf]

Supplementary Online Content

Lyhne JD, Smith AB, Timm S, et al. E-Health intervention for fear of cancer recurrence: a randomized clinical trial. *JAMA Netw Open*. 2025;8(11):e2542112.
doi:10.1001/jamanetworkopen.2025.42112

eTable 1. Change in FCRI Triggers Subscale Between Baseline (T0) and Postintervention (T1) and 3- and 6-Month Follow-Up (T2 and T3)

eTable 2. Change in FCRI Psychological Distress Subscale Between Baseline (T0) and Postintervention (T1) and 3- and 6-Month Follow-Up (T2 and T3)

eTable 3. Change in FCRI Functional Impairment Subscale Between Baseline (T0) and Postintervention (T1) and 3- and 6-Month Follow-Up (T2 and T3)

eTable 4. Change in FCRI Insight Subscale Between Baseline (T0) and Postintervention (T1) and 3- and 6-Month Follow-Up (T2 and T3)

eTable 5. Change in FCRI Reassurance Subscale Between Baseline (T0) and Postintervention (T1) and 3- and 6-Month Follow-Up (T2 and T3)

eTable 6. Change in FCRI Coping Strategies Subscale Between Baseline (T0) and Postintervention (T1) and 3- and 6-Month Follow-Up (T2 and T3)

eTable 7. Process Measures: Change in Outcome During Intervention Compared to Baseline

eTable 8. Characteristics of Completers Compared to Noncompleters

This supplementary material has been provided by the authors to give readers additional information about their work.

© 2025 Lyhne JD et al. *JAMA Network Open*.

eTable 1. Change in FCRI triggers subscale between baseline (T0) and post-intervention (T1) and 3- and 6-month follow-up (T2 and T3)				
	Triggers (scale score 0-32)			
Time point<br>Mean (sd), *n*	Intervention	Augmented control	Difference between groups (95% CI)*	*p*
T0 (Baseline)	16.2 (5.1), 42	16.0 (6.1), 53		
T1 (Post-treatment)	17.6 (5.7), 29	18.6 (5.1), 41	2.8 (0.5 - 5.1)	0.0176
Change (T0 – T1)				
T2 (3 month FU)	15.9 (6.0), 27	18.0 (5.7), 42	3.9 (1.2 - 6.6)	0.0060
Change (T0 – T2)				
T3 (6 month FU)	14.8 (6.5), 25	15.7 (5.2), 32	4.2 (1.4 - 7.0)	0.0035
Change (T0 – T3)				
* Change score is based on complete matched data				

© 2025 Lyhne JD et al. *JAMA Network Open*.

eTable 2. Change in FCRI psychological distress subscale between baseline (T0) and post-intervention (T1) and 3- and 6-month follow-up (T2 and T3)

	Psychological distress (scale score 0-16)			
Time point<br>Mean (sd), *n*	Intervention	Augmented control	Difference between groups (95% CI)*	*p*
T0 (Baseline)	8.1 (4.2), 42	7.7 (3.3), 53		
T1 (Post-treatment)	5.1 (3.6), 29	8.7 (2.6), 41	4.2 (2.5 - 5.9)	0.0000
Change (T0 – T1)				
T2 (3 month FU)	5.1 (3.4), 27	7.8 (3.1), 42	4.3 (1.0 - 7.6)	0.0113
Change (T0 – T2)				
T3 (6 month FU)	4.0 (3.0), 25	6.8 (3.2), 32	3.9 (0.6 - 7.2)	0.0222
Change (T0 – T3)				

* Change score is based on complete matched data

© 2025 Lyhne JD et al. *JAMA Network Open*.

**eTable 3. Change in FCRI functional impairment subscale between baseline (T0) and post-intervention (T1) and 3- and 6-month follow-up (T2 and T3)**				
	Functional Impairments (scale score 0-24)			
Time point<br>Mean (sd), *n*	Intervention	Augmented control	Difference between groups (95% CI)*	*p*
T0 (Baseline)	9.4 (6.1), 42	9.5 (5.2), 53		
T1 (Post-treatment)	6.2 (5.3), 29	9.2 (4.7), 40	3.4 (1.1 - 5.8)	0.0044
Change (T0 – T1)				
T2 (3 month FU)	6.9 (6.1), 27	8.8 (5.8), 42	2.5 (-0.3 - 5.3)	0.0825
Change (T0 – T2)				
T3 (6 month FU)	6.0 (5.9), 25	7.5 (5.2), 32	3.1 (0.7 - 5.5)	0.0125
Change (T0 – T3)				
* Change score is based on complete matched data				

© 2025 Lyhne JD et al. *JAMA Network Open*.

**eTable 4. Change in FCRI insight subscale between baseline (T0) and post-intervention (T1) and 3- and 6-month follow-up (T2 and T3)**				
	Insight (scale score 0-12)			
Time point<br>Mean (sd), *n*	Intervention	Augmented control	Difference between groups (95% CI)*	*p*
T0 (Baseline)	4.0 (2.7), 42	4.3 (2.4), 53		
T1 (Post-treatment)	2.0 (1.8), 29	4.0 (2.2), 40	2.2 (1.1 - 3.3)	0.0001
Change (T0 – T1)				
T2 (3 month FU)	2.1 (2.4), 27	4.2 (2.5), 42	1.9 (-0.9 - 4.7)	0.1841
Change (T0 – T2)				
T3 (6 month FU)	1.9 (2.4), 25	3.8 (2.6), 32	2.0 (0.6 - 3.3)	0.0048
Change (T0 – T3)				
* Change score is based on complete matched data				

© 2025 Lyhne JD et al. *JAMA Network Open*.

**eTable 5. Change in FCRI reassurance subscale between baseline (T0) and post-intervention (T1) and 3- and 6-month follow-up (T2 and T3)**				
	Reassurance (scale score (0-12)			
Time point<br>Mean (sd), *n*	Intervention	Augmented control	Difference between groups (95% CI)	*p*
T0 (Baseline)	2.2 (2.0), 42	3.0 (2.4), 53		
T1 (Post-treatment)	2.0 (1.9), 29	3.5 (2.3), 39	0.6 (-0.4 - 1.6)	0.2124
Change (T0 – T1)				
T2 (3 month FU)	2.8 (2.0), 27	3.0 (2.1), 42	-0.4 (-1.3 - 0.6)	0.4622
Change (T0 – T2)				
T3 (6 month FU)	2.9 (2.5), 25	2.8 (2.2), 32	-0.2 (-1.6 - 1.1)	0.7512
Change (T0 – T3)				
* Change score is based on complete matched data				

© 2025 Lyhne JD et al. *JAMA Network Open*.

eTable 6. Change in FCRI coping strategies subscale between baseline (T0) and post-intervention (T1) and 3- and 6-month follow-up (T2 and T3)

	Coping strategies (scale score 0-36)			
Time point<br>Mean (sd), *n*	Intervention	Augmented control	Difference between groups (95% CI)*	*p*
T0 (Baseline)	19.3 (6.0), 42	19.3 (5.8), 53		
T1 (Post-treatment)	19.7 (5.6), 29	18.9 (5.1), 39	0.0 (-2.0 - 2.0)	0.9775
Change (T0 – T1)				
T2 (3 month FU)	17.7 (7.0), 27	17.3 (5.4), 42	1.3 (-0.9 - 3.5)	0.2374
Change (T0 – T2)				
T3 (6 month FU)	17.7 (6.6), 25	16.9 (5.5), 32	1.1 (-1.3 - 3.5)	0.3466
Change (T0 – T3)				

* Change score is based on complete matched data

eTable 7. Process measures: Change in outcome during intervention compared to baseline						
**Outcome (score range)**	T0 Mean (sd) *n, p*	M3 Mean (sd) *n, p*	M5 Mean (sd) *n, p*	T1 Mean (sd) *n, p*	T2 Mean (sd) *n, p*	T3 Mean (sd) *n, p*
**Negative beliefs about worry (6-24)**	12.0 (3.7)<br>42	12.0 (3.0)<br>27, 0.71	10.1 (2.7)<br>25, 0.01	10.2 (3.4)<br>29, 0.05	10.3 (3.8)<br>27, 0.04	9.8 (2.9)<br>25, 0.02
**Perceived risk of recurrence (1-100)**	55.7 (18.2)<br>42	55.4 (25.8)<br>15, 0.30	42.3 (23.0)<br>16, 0.03	36.1 (22.1)<br>29, >0.01	40.3 (21.6)<br>27, >0.01	37.0 (26.2)<br>25, >0.01
M3: Module 3 at intervention week four; M5: Module 5 at intervention week eight						

© 2025 Lyhne JD et al. *JAMA Network Open*.

eTable 8. Characteristics of completers compared to noncompleters			
**Variable (%)**	Completers (*n*=23)	Non-completers (*n*=19)	*p*
Age	64 (9.8) 50-81	66 (8.6) 44-79	0.3482
Sex, female	16 (69.6)	11 (57.9)	0.432
Education			0.118
Short	2 (9.1)	5 (26.3)	
Medium	10 (45.5)	3 (15.8)	
Long	10 (45.5)	10 (52.6)	
Other	0	1 (5.3)	
**Data from T0, mean (sd)**			
FCRI-SF score	25.3 (3.0)	25.5 (3.0)	0.8479
FCRI total score	87.3 (21.4)	83.8 (16.5)	0.8088
Anxiety score	5.7 (4.0)	3.9 (2.5)	0.1084
Depression score	5.7 (5.6)	4.3 (3.1)	0.3506
Distress score	11.0 (6.9)	7.7 (4.6)	0.0899
Physical symptom score	35.8 (20.8)	27.1 (12.5)	0.1407
Quality of life	66.8 (19.6)	54.1 (19.7)	0.0431

© 2025 Lyhne JD et al. *JAMA Network Open*.

Motivation	8.6 (1.2)	7.8 (2.8)	0.2116
Number of messages per module	2.7 (1.0)	2.9 (1.4)	0.6814
Words per message	149 (36.1)	127 (36.0)	0.0492
**Data from T0, mean (sd)**			
FCRI total score	67.4 (21.3)	79.4 (36.6)*	0.33
Quality of life	69.5 (16.9)	70.0 (21.3)*	0.9548
*Data from five participants available			

© 2025 Lyhne JD et al. *JAMA Network Open*.
