# Supplementary material for: E-Health Intervention for Fear of Cancer Recurrence: A Randomized Clinical Trial
Source: JAMA Netw Open. 2025 Nov 11;8(11):e2542112. doi: 10.1001/jamanetworkopen.2025.42112 (PMC12606383; doi:10.1001/jamanetworkopen.2025.42112)
Supplement: Supplement 3. — Data Sharing Statement [file jamanetwopen-e2542112-s003.pdf]

Data Sharing Statement

Lyhne. E-Health Intervention for Fear of Cancer Recurrence. *JAMA Netw Open*. Published November 11, 2025. doi:10.1001/jamanetworkopen.2025.42112

Data
Additional Information: https://clinicaltrials.gov/ ClinicalTrials.gov: NCT04287218
Data available: Yes
Data types: Deidentified participant data
How to access data: The datasets generated during and/or analyzed during the current study are available from the corresponding author on reasonable request (Johanne.Dam.Lyhne@rsyd.dk)
When available: With publication

Supporting Documents
Document types: Statistical/analytic code
How to access documents: The statistical code generated during the current study are available from the corresponding author on reasonable request (Johanne.Dam.Lyhne@rsyd.dk)
When available: With publication

Additional Information
Who can access the data: Data will be made available for researchers whose proposed use of the data has been approved
Types of analyses: The data will be made available for a specified purpose
Mechanisms of data availability: The data will be made available with investigator support
